# Effects of parental overweight and obesity on offspring’s mental health: A meta-analysis of observational studies

**DOI:** 10.1371/journal.pone.0276469

**Published:** 2022-12-22

**Authors:** Shuyu Zhang, Tingting Lin, Yu Zhang, Xinmei Liu, Hefeng Huang

**Affiliations:** 1 The International Peace Maternity and Child Health Hospital, School of Medicine, Shanghai Jiao Tong University, Shanghai, China; 2 Shanghai Key Laboratory of Embryo Original Diseases, Shanghai, China; 3 School of Nursing, Hangzhou Medical College, Hangzhou, China; 4 Obstetrics and Gynecology Hospital, Institute of Reproduction and Development, Fudan University, Shanghai, China; 5 Research Units of Embryo Original Diseases, Chinese Academy of Medical Sciences (No. 2019RU056), Shanghai, China; 6 Key Laboratory of Reproductive Genetics (Ministry of Education), Department of Reproductive Endocrinology, Women’s Hospital, Zhejiang University School of Medicine, Hangzhou, China; Endocrinology and Metabolism Population Sciences Institute, Tehran University of Medical Sciences, ISLAMIC REPUBLIC OF IRAN

## Abstract

**Background:**

Children of parents who were overweight/obese prior to pregnancy face a variety of neurodevelopmental challenges. The goal of this meta-analysis is to compile evidence about the impact of parental overweight/obesity on their children’s mental health.

**Methods:**

The databases Cochrane Library, EMBASE, Pubmed, PsycINFO, and Web of Science were searched until May 2022. The pooled effect size was calculated using the fixed and random effect models. We also performed I^2^ index, subgroup analyses, sensitivity analyses, quality assessment, and publication bias analysis. The protocol was registered on the PROSPERO database (CRD42022334408).

**Results:**

For maternal exposure (35 studies), both maternal overweight [OR 1.14 (95% CI 1.10,1.18)] and maternal obesity [OR 1.39 (95% CI (1.33, 1.45)] were significantly associated with offspring’s mental disorders. Maternal pre-pregnancy overweight/obesity increased the risk of attention-deficit/hyperactivity disorder (ADHD) [OR 1.55 (95% CI 1.42,1.70)], autism spectrum disorder (ASD) [OR 1.37 (95% CI 1.22,1.55)], cognitive/intellectual delay [OR 1.40 (95% CI 1.21,1.63)], behavioral problems [OR 1.50 (95% CI 1.35,1.66)] and other mental diseases [OR 1.30 (95% CI 1.23,1.37)]. For paternal exposure (6 studies), paternal obesity [OR 1.17 (95% CI 1.06, 1.30)] but not overweight [OR 1.03 (95% CI 0.95,1.11)] was significantly associated with offspring’s mental disorders.

**Conclusions:**

Parental overweight/obesity might have negative consequences on offspring’s mental health and pre-pregnancy weight control is advised.

## Introduction

The prevalence of mental illness is clearly on the rise. According to the Global Burden of Diseases 2019 report, there is no global indication of a decline in mental illnesses since 1990, placing them among the top 10 primary sources of burden globally [1]. A diverse set of genetic and/or environmental risk factors play an important role in the etiology of mental illness by altering brain structure and/or function. Notably, the incidence of parental obesity has been rising together with the prevalence of mental illness, suggesting a possible link between the two phenomena [2]. Studies have shown children born to obese mothers may have a higher risk of ASD and ADHD, as well as perform worse in terms of intellectual disability (ID) than children born to normal weight mothers. Paternal obesity may also negatively impact cognitive and behavioral outcomes in offspring [3–5]. The underlying mechanisms causing these later-life detrimental consequences in offspring are not clear. Potential pathways whereby metabolized nutritional components may affect child development. Both maternal and paternal obesity can impact several processes involved in the conception, as well as intrauterine and postnatal development of the offspring [3, 6].

Previous meta-analysis established a link between maternal obesity and offspring’s neurodevelopment. All of them reported offspring exposed to maternal overweight/obesity, might raise the risk of psychiatric disorders such as ADHD, ASD, negative emotion, schizophrenia, and behavior problems [7–12]. But they seldom comprehensively assessed their offspring’s mental health and failed to carefully check maternal weight exposure prior to pregnancy. Despite the equal prevalence of obesity in both sexes [13–15], the vast majority of research has focused on maternal nutritional influences on children during gestation and lactation. However, limited attention has been paid to the effect of paternal weight on offspring [16]. None of the previous meta analyses considered the effect of paternal overweight/obesity on offspring’s mental health. Only one analyzed the relationship between paternal body mass index (BMI) and ASD in the offspring [9] and stated that paternal BMI has no association with offspring’s ASD because of insufficient evidence. More importantly, a large number of studies on this topic have been published in recent years and have explored the effect of paternal BMI, with inconsistent outcomes [17–19].To better summarize the expanding research, we performed a meta-analysis to fully evaluate the association between parental obesity/overweight and offspring’s mental health.

## Methods

We adhered to the Preferred Reporting Items for Systematic Reviews and Meta-Analyses guidelines [20] and registered our meta-analysis on PROSPERO(CRD42022334408).

### Search strategy

Cochrane Library, EMBASE, Pubmed, PsycINFO and Web of Science databases were searched up to May 2022. Medical Subject Headings (MeSH), Emtree Headings and other relevant key words were used to find studies related to parental BMI and the risk of mental diseases in offspring. We searched using the following general terms: (parent) AND (BMI) AND (offspring) AND (mental illness) AND (case–control OR cohort study). We used truncations and wildcards to accommodate synonyms and variations. Additionally, we looked for further studies by manually scanning review papers that came up in our searches. Two authors (Zhang and Lin) checked reference lists to find research that would be possibly eligible for our inclusion criteria. The full search strategy can be found in S1 Table.

### Study selection

Two authors screened records separately. First, titles and abstracts of articles were examined and full texts would be retrieved if necessary. Inclusion criteria:(1) Population: parents and their offspring;(2) Exposure: evaluated either the paternal or maternal pre-pregnancy weight status;(3) Outcome: addressed the mental health of offspring;(4) Study design: original observational study. Exclusion criteria:(1) Target population involved parents with mental disorder or children not born at full term;(2) The parental BMI used was not measured at pre-pregnancy; (3) The study reported continuous outcome data. Disagreements were resolved in a group discussion.

### Data extraction

Data extraction was completed by two investigators (Zhang and Lin). The following information was extracted: (1) Basic research information about authorship and date of publication; (2) Cohort characteristics included study design, country, sample size, cohort name and covariates; (3) Exposure information included parental weight status and BMI criteria; (4) Outcome information included the offspring’s age at evaluation, neurodevelopmental outcomes and their diagnostic methods (5) Odds Ratios(OR), Risk Ratio (RR), or Hazard Ratio(HR) with corresponding 95% confidence interval (CI) was also extracted from each included study.

### Quality assessment

The Newcastle–Ottawa Quality Assessment Scale (NOS) was used to assess the quality of the included studies, which is recommended by Cochrane Collaboration [21]. We regarded studied as high quality when they had 6 or more stars.

### Statistical analysis

In this meta-analysis, the following issues with repeated measurements in the same sample should be taken into account: (1) Data from studies with two cohorts were analyzed as independent samples; (2) The meta-analysis only included studies reporting separate data for pre-pregnancy overweight and obesity;(3)When neurodevelopment was examined using several tests within the same cohort, we took into account the values for all of the tests and if a test provided a total score value, this was the only value taken into account for the pooled estimate; (4) When several estimates were reported within the same study, the most adjusted model was used for the pooled estimate.

For all outcomes, estimates were converted into OR (where possible) and pooled OR were estimated using the fixed and random effect models. Heterogeneity was assessed using the I^2^ statistic. I^2^ values of <25%, 25–50% and >50% usually correspond to small, medium and large heterogeneity, respectively [22].

The following subgroup analyses were conducted: (1) Analyze the relationship between parental BMI pre-pregnancy categories and the risk of mental illness in offspring; (2) Analyze the relationship between maternal BMI pre-pregnancy categories and the risk of specific outcomes (ADHD, ASD, cognitive/intellectual delay, behavioral problems, and other mental diseases including schizophrenia, negative emotions, psychomotor development, etc.). The presence of publication bias was quantified using the Begg’s test and Egger’s test.

We performed three kinds of sensitivity analyses to examine the robustness of our results:(1) We defined higher-quality studies as those that had high scores on the quality measure;(2) We grouped studies by their study design;(3) We removed studies one by one to assess the robustness of the summary estimates. All statistical analyses were conducted using Stata, version 16.0 (Stata Corp, College Station, TX, USA).

## Results

### Literature search

**Fig 1:**
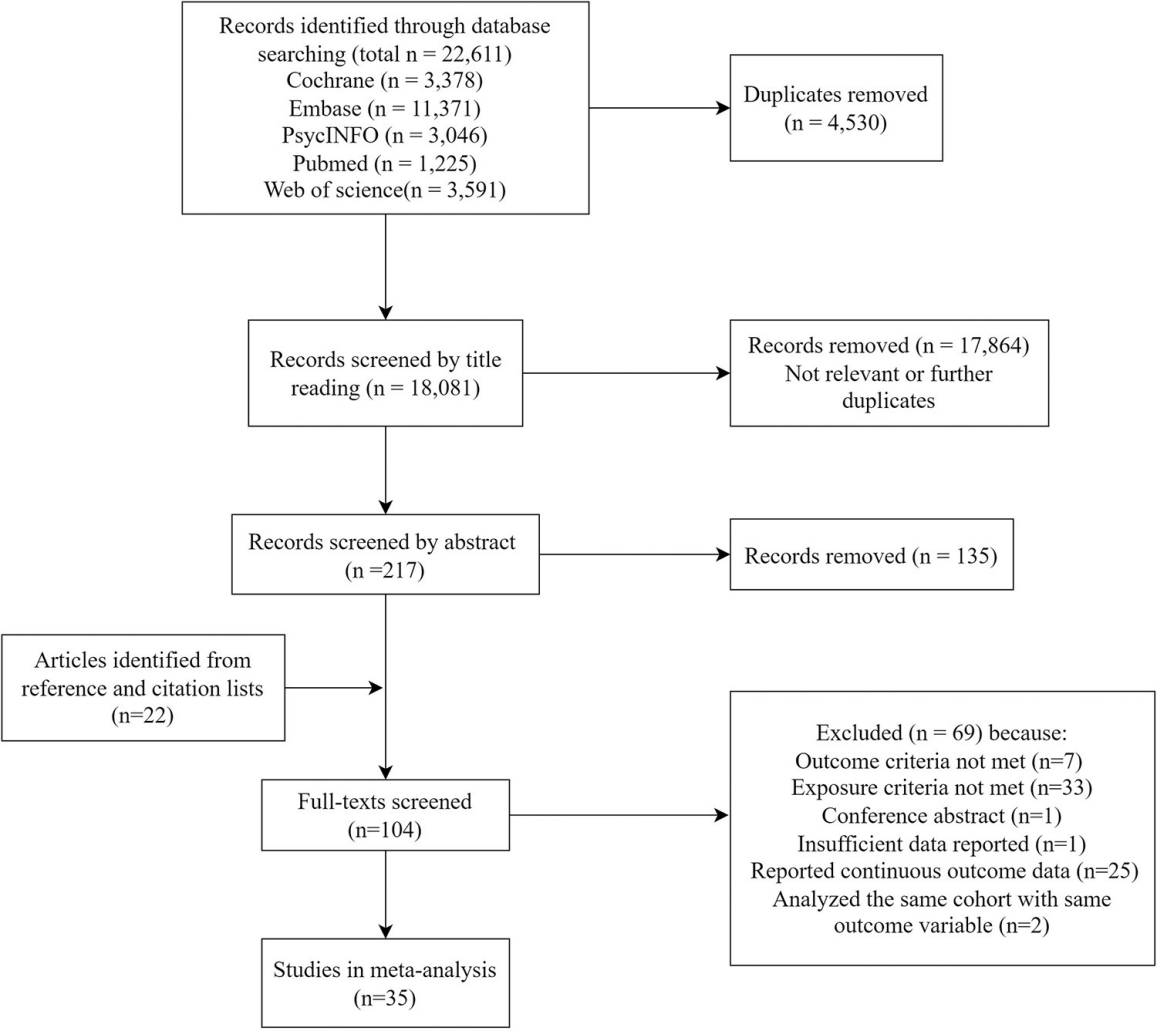
Study selection flowchart.

A total of 22 611 records were identified in the above databases (Fig 1). After removing duplicates (4 530), 18 081 records were yielded. Of these, most records were removed from the title (n = 17 864) and abstract screening (135) because they were non-relevant study designs or further duplicates. Besides, 22 additional articles were found during manual searching from the reference lists of relevant records. Thus, 104 articles were considered for full-text screening, and 69 were excluded, giving final 35 studies included in the meta-analysis.

### Study characteristics

Of the 35 included studies, 24 were prospective studies [23–46], 6 were retrospective studies [47–52]and 5 were case–control studies [53–57] (Table 1). Note that 4 articles included in their analysis two cohorts. The included samples ranged from 197 to 620 795, belonging to 39 cohorts started between 1959 and 2008. For the geographical area of included studies, 18 studies were from the America, 14 from Europe, 2 from Australasia and 1 from Asia. There were 30 high quality studies and 5 low quality studies.

**Table 1:** Descriptive summary of the included 35 studies.

General character	No. of studies (%)
**Study design**	
Prospective	24 (68.6%)
Retrospective	6 (17.1%)
Case–Control	5 (14.3%)
Sample size	
<1000	3 (8%)
1000–5000	16 (46%)
>5000	16 (46%)
Origin of sample	
Australasia	2 (6%)
Asia	1 (3%)
Europe	14 (40%)
America	18 (51%)
Quality grade of study	
High (NOS≥6)	30 (86%)
Low (NOS<6)	5 (14%)
**Exposure character**	
Maternal exposure	
Overweight	31 (89%)
Obesity	32 (91%)
Overweight/Obesity	3 (9%)
Paternal exposure	
Overweight	4 (11%)
Obesity	5 (14%)
Overweight/Obesity	1 (3%)
**Outcome character**	
ADHD	10 (29%)
ASD	15 (43%)
Cognitive/intellectual delay	7 (20%)
Behavioral problems	9 (26%)
Other mental diseases	14 (40%)

**Note:** NOS, Newcastle–Ottawa Quality Assessment Scale; ADHD, Attention-Deficit/Hyperactivity Disorder; ASD, Autism Spectrum Disorder.

All studies assessed the exposure to maternal prepregnant overweight and obesity, while six studies also investigated the exposure to fathers. All but 8 studies [29, 30, 34, 37, 38, 41, 53, 54] defined weight groups according to World Health Organization categories(WHO).4 articles [24, 32, 35, 38] collapsed underweight and normal weight into the same category (BMI < 24.99), which was analyzed collectively as normal weight,9 studies [24, 32, 39, 40, 46, 49–51, 54] further divided obesity into Obese Class I, II and III (or II/III), 3 articles [44, 45, 53] grouped overweight and obese mothers together. Thus, these were analyzed only as a combined obese and overweight category. Of the 35 studies reporting maternal exposure, 31 involved maternal overweight and 32 involved maternal obesity. Among the 6 studies [24, 28, 32, 35, 43, 44] reported paternal exposure, 4 examined paternal overweight [28, 32, 35, 43] and 5 examined paternal obesity [24, 28, 32, 35, 43]. Different diagnostic methods were used to determine each mental health outcome (S2 Table). Studies reporting maternal exposure were further grouped into the categories according to offspring outcomes: ADHD (n = 10), ASD (n = 15), cognitive and intellectual development(n = 7), behavioral problems (n = 9) and other mental diseases (n = 14). Table 2 contains summary characteristics for all studies included in meta-analyses.

**Table 2:** Characteristics of the included 35 studies.

Study	Design; Country(start year);n	Cohort name	Parent exposure	BMI standard(year); categories	BMI source	Offspring’s outcome(age)	Adjusted covariates	NOS score
Schaefer et al. (2000) [29]	Prospective study; USA(1959); 6,633	Child Health and Development Study	Mother	Institute of Medicine (1990); low ≤19.9 average 20.0–26.9 above average 27.0–29.9 high ≥30.0	Medical record	Other mental diseases: schizophrenia spectrum disorder (adolescent and adult)	Maternal age, parity, education, race, cigarette smoking, and gender of the offspring	8
Heikura et al. (2008) [41]	Prospective study; Finland(1966), Finland(1986) 12,058; 9,432	Northern Finland Birth Cohorts	Mother	NR; thin <20.0 normal 20.0–24.9 overweight 25.0–29.9 obese ≥30.0	Self-report and medical staff	Cognitive/intellectual delay (<11.5y)	Age, smoking, maternal education, parity and family structure, marital status, place of residence, number of visits to maternity health center	5
Rodriguez et al. (2008) [30]	Prospective study; Sweden(2001) Denmark(2001) Finland(1993); 12,556	Nordic Network on ADHD	Mother	NR; underweight<18 normal 19–26 overweight>26	Medical record and questionnaire	ADHD(7-12y)	Maternal smoking, weight gain, gestational age, birth weight, infant sex, maternal age, maternal education and family structure	6
Rodriguez et al. (2010) [31]	Prospective study; Sweden(1999); 1,009	Pregnancy Cohort from Sweden	Mother	WHO(NR); underweight <18.5 normal 18.50–24.99 overweight 25.00–29.99 obese ≥30.00	Medical record	ADHD; other mental diseases: negative emotion; behavioral problems (5y)	Maternal smoking during pregnancy, maternal education, maternal age, gestational age, birth weight, infant sex, family structure at follow-up, family structure, depressive symptoms, life events and current: child overweight, parental ADHD symptoms, and maternal depressive symptoms	8
Brion et al. (2011) [44]	Prospective study; British(1991),Dutch(2002); 4,712, 2,046	British Avon Longitudinal Study of Parents and Children; Dutch Generation R	Mother; father	WHO(NR); underweight <18.5 normal 18.50–24.99 overweight 25.00–29.99 obese ≥30.00	Self-report	Behavioral problems; cognitive/intellectual delay (3.2–3.9y;4.9–8.0y)	Maternal education, paternal education, family income, social class, and maternal smoking.	7
Lyall et al. (2011) [37]	Prospective study; USA(1989); 61,596	Nurses’ Health Study II	Mother	NR; Underweight <22 normal 22–25 overweight 25–30 obesity ≥30	Self-report	ASD(NR)	Age at baseline, race, income; BMI, body shape at age 20, and cycle length and regularity models also included age at menarche	5
Hinkle et al. (2012) [51]	Retrospective cohort study; USA(2001) 6,850	Early Childhood Longitudinal Study Birth Cohort	Mother	WHO(1998); underweight <18.5 normal 18.50–24.99 overweight 25.00–29.99 obese class I 30.0–34.9 obese class II 35.0–39.9 class III ≥40.0	Self-report	Cognitive/intellectual delay; other mental diseases: psychomotor development(0.8–2y)	Maternal age (continuous), race-ethnicity, marital status, parity, schooling (continuous), smoking during pregnancy, household poverty status and child sex	7
Krakowiak et al. (2012) [55]	Case-control study; USA (2003); 1,004	Childhood Autism Risks from Genetics and the Environment	Mother	WHO(NR); underweight <18.5 normal 18.50–24.99 overweight 25.00–29.99 obese ≥30.00	Medical record and medical staff	ASD; other mental diseases: DD without ASD(2-5y)	Mother’s age at delivery, race/ethnicity, education level, delivery payer, calendar time, child’s age at enrollment and gender, and catchment area	8
Hinkle et al. (2013) [50]	Retrospective cohort study; USA (2001); 5,200	Early Childhood Longitudinal Study Birth Cohort	Mother	WHO(2000); underweight <18.5 normal 18.50–24.99 overweight 25.00–29.99 obese class I 30.0–34.9 obese class II and III ≥35.0	Self-report	Behavioral problems, other mental diseases: psychomotor development (4.8–7.1y)	Demographics, smoking, enrichment and current child weight status	7
Mann et al. (2013) [49]	Retrospective cohort study; USA(2004); 78,675	South Carolina Medicaid Programme	Mother	WHO(NR); underweight <18.5 normal 18.50–24.99 overweight 25.00–29.99 obese class I 30.0–34.9 obese class II 35.0–39.9 class III ≥40.0	Self-report	Cognitive/intellectual delay (3–6y)	Maternal age, race, ethnicity, and level of education, maternal tobacco use, gonorrhea, chlamydia, syphilis, intrapartum fever, epilepsy, hypertension, diabetes mellitus, child’s sex, gestational age at delivery, and birthweight	7
Robinson et al. (2013) [33]	Prospective study; Australian(1989); 2,765	Western Australian Pregnancy Cohort (Raine)	Mother	WHO(2004); underweight <18.5 normal 18.50–24.99 overweight 25.00–29.99 obese ≥30.00	Self-report and research staff	Other mental diseases: affective problems (5–17y)	Maternal age at conception, maternal education, total family income, maternal smoking in pregnancy, maternal alcohol consumption in pregnancy, the presence of the biological father in the family home, the maternal experience of stressful events and perinatal data	6
Antoniou et al. (2014) [45]	Prospective study; UK (2008); 788	Twins and Multiple Birth Association Heritability Study	Mother	WHO(NR); underweight <18.5 normal 18.50–24.99 overweight 25.00–29.99 obese ≥30.00	Self-report	Behavioral problems (1.5–5y)	Gestational age, maternal educational level and smoking, twins‘ age, sex, birth weight	5
Chen et al. (2014) [42]	Prospective study; Sweden(1973); 620,795	Sweden population-based cohort	Mother	WHO(NR); underweight <18.5 normal 18.50–24.99 overweight 25.00–29.99 obese ≥30.00	Medical record	ADHD(>3y)	Offspring sex, birth order, year of birth, mother’s country of birth, maternal education, maternal age at delivery, smoking during pregnancy, cohabitation with child’s father at childbirth	8
Moss et al. (2014) [48]	Retrospective cohort study; USA (2001); 4,800	Early Childhood Longitudinal Study Birth Cohort	Mother	WHO(1995); underweight <18.5 normal 18.50–24.99 overweight 25.00–29.99 obese ≥30.00	Self-report	ASD(0.8–2y)	Maternal age, child sex, birthweight, rates of height growth and weight gain	5
Surén et al. (2014) [28]	Prospective study; Norway (1999); 50,116	Norwegian Mother and Child Cohort	Mother, father	WHO(2013); underweight <18.5 normal 18.50–24.99 overweight 25.00–29.99 obese ≥30.00	Self-report	ASD(4–13.1y)	Parental education, parental age, parental smoking, parental psychiatric disorders, maternal parity, maternal use of folic acid supplements, use of hormone treatment or in vitro fertilization to become pregnant, maternal diabetes, preeclampsia, child’s year of birth, child’s gestational age at birth, and child’s birth weight	9
Tanda et al. (2014) [27]	Prospective study; USA (1976); 2,127(White); 1,268(African-American)	National Longitudinal Survey of Youth	Mother	WHO(NR); underweight <18.5 normal 18.50–24.99 overweight 25.00–29.99 obese ≥30.00	Self-report	Behavioral problems (8–9.2y)	Child’s birth weight, gestational age, and mother’s smoking during pregnancy, maternal education, child’s home environment, household income, and marital status of their mothers, child’s race, gender, birth order, child’s age in month, and child’s weight status	5
Jo et al. (2015) [40]	Prospective study; USA (2005); 1,311	Infant Feeding Practices Study II	Mother	WHO(2000); underweight <18.5 normal 18.50–24.99 overweight 25.00–29.99 obese class I 30.0–34.9 obese class II 35.0–39.9 class III ≥40.0	Self-report	ADHD; ASD; behavioral problems; cognitive/intellectual delay; other mental diseases: depression or anxiety, motor skills (6y)	Maternal age, maternal race or ethnicity, marital status, mother’s education, poverty-to-income ratio, smoking during third trimester, parity, child’s gender, child’s current BMI, and child’s enrichment, birth weight, pregnancy weight gain, gestational diabetes, breastfeeding, and postpartum depression.	8
Torres-Espinola et al. (2015) [26]	Prospective study; Spain(2007) 215; 197	PREOBE Cohort	Mother	WHO(NR); underweight <18.5 normal 18.50–24.99 overweight 25.00–29.99 obese ≥30.00	Medical record and questionnaire	Cognitive/intellectual delay; other mental diseases: psychomotor development, composite socio emotion (0.5y,1.5y)	Maternal age, maternal educational level, placental weight, and weight gain during pregnancy	8
Xiang et al. (2015) [47]	Retrospective cohort study; USA (1995); 68,837	Kaiser Permanente Southern California longitudinal cohort	Mother	WHO(NR); underweight <18.5 normal 18.50–24.99 overweight 25.00–29.99 obese ≥30.00	Medical records	ASD(1.5 and 2y)	Maternal age at delivery, parity, education, self-reported maternal race/ethnicity, median family household income based on census tract of residence, history of comorbidity, and sex of the child	9
Connolly et al. (2016) [57]	Case–Control Study; USA (2006); 39,313	Cincinnati Children’s Hospital Medical Center’s Cohort	Mother	WHO(NR); underweight <18.5 normal 18.50–24.99 overweight 25.00–29.99 obese ≥30.00	Medical records	ASD(5.5y)	Maternal age at birth, maternal race, year of birth	7
Getz et al. (2016) [56]	Case–Control Study; UK (1993); 4,419	General Practice Research Database	Mother	WHO(NR); underweight <18.5 normal 18.50–24.99 overweight 25.00–29.99 obese ≥30.00	Medical records	ASD(6.2y)	Maternal age (continuous), maternal pre-pregnancy depression, diabetes, smoking status, drug abuse, alcoholism, in addition to matching factors (birth year, sex, and general practice)	6
Li et al. (2016) [38]	Prospective study; USA (1998); 1,767	Boston Birth Cohort	Mother	CDC(2014); underweight /normal<25.0 overweight 25.00–29.99 obese ≥30.00	Self-report	ADHD; ASD; cognitive/intellectual delay; other mental diseases: DD(3.6–9.0y)	Child year of birth, child gender, maternal age, parity, smoking during pregnancy and preterm birth	8
Casas et al. (2017) [43]	Prospective study; Spanish(2003); 1,827	INfancia y Medio Ambiente—Environment and Childhood	Mother, father	WHO(NR); underweight <18.5 normal 18.50–24.99 overweight 25.00–29.99 obese ≥30.00	Self-report	ADHD; ASD (mean:4.8 y)	Age and sex of the child, maternal and paternal education and social class, maternal age, parity, maternal employment status during pregnancy and at 5 years, maternal IQ, breast feeding duration, daycare attendance, and child physical activity paternal or maternal BMI	7
Mikkelsen et al. (2017) [35]	Prospective study; Denmark(1996); 38,314	Danish National Birth Cohort	Mother, father	WHO(2000); underweight/normal (<25.0) overweight 25.00–29.99 obese ≥30.00	Self-report	Behavioral problems(7y)	Parental age, marital status, socioeconomic status, smoking, parity, birth year, sex, parental BMI, and parental hyperactivity	7
Yeung et al. (2017) [24]	Prospective study; USA (2008); 4,821	Upstate KIDS Study	Mother, father	WHO(NR); underweight/normal (<25.1) overweight 25.00–29.99 obese class I 30.0–34.9 obese class II 35.0–39.9 class III ≥40.0	Medical records and self-report	Other mental diseases: DD(0.3-3y)	Maternal age, race, education, insurance, married, previous live birth, pregnancy smoking and parent BMI	7
Andersen et al. (2018) [46]	Prospective study; Denmark(1996); 81,892	Danish National Birth Cohort	Mother	WHO(1993); underweight <18.5 normal 18.50–24.99 overweight 25.00–29.99 obese class I 30.0–34.9 obese class II 35.0–39.9 class III ≥40.0	Self-report	ADHD;ASD(mean 13.3 y)	Socioeconomic status, maternal smoking, maternal psychiatric diagnoses, parental age, gestational age and birth weight	9
Menting et al. (2018) [36]	Prospective cohort study; Netherlands(2003); 4,094	Amsterdam Born Children and their Development	Mother	WHO(NR); underweight <18.5 normal 18.50–24.99 overweight 25.00–29.99 obese ≥30.00	Self-report	Behavioral problems (5-7y)	Sex and age of the child, maternal age, maternal ethnicity, maternal educational level and parity, maternal mental health disorder, paternal mental health disorder, smoking during pregnancy, alcohol use during pregnancy and child BMI z-score	7
Shen et al. (2018) [53]	Case–Control Study; China(NR); 2,897	Elim Training Center for Children with Autism	Mother	BMI classification standards for the Chinese population(2002); underweight <18.5 normal 18.5–24.0 overweight 24.0–28.0 obese ≥28.0	Self-report	ASD(2–9y)	Child’s gender, child age, parental age, maternal history of alcoholism/drug use during pregnancy, family annual income	7
Grudzinski et al. (2019) [52]	Retrospective cohort study; Canada(1989); 38,211	Nova Scotia population-based cohort	Mother	WHO(2000); underweight <18.5 normal 18.50–24.99 overweight 25.00–29.99 obese ≥30.00	Medical records	ADHD; other mental diseases: any mental health disorder(0-18y)	Maternal age, area of residence, area-level income quintile, marital status, parity, maternal psychiatric disorders during pregnancy, and smoking in pregnancy	7
Varcin et al. (2019) [25]	Prospective study; Australian(1989); 1,238	Western Australian Pregnancy Cohort (Raine) Study	Mother	WHO(2000); underweight <18.5 normal 18.50–24.99 overweight 25.00–29.99 obese ≥30.00	Self-report and research staff	ASD(19-20y)	Paternal obesity at time of pregnancy, maternal age at conception, maternal smoking during pregnancy, alcohol consumption during pregnancy, maternal hypertensive diseases of pregnancy, maternal education, family income at time of pregnancy, threatened abortion, offspring gender, diabetes, and parity	7
Robinson et al. (2020) [32]	Prospective cohort study; USA (2008); 2,870	Upstate KIDS	Mother, father	WHO(NR); underweight/normal <25.1 overweight 25.00–29.99 obese class I 30.0–34.9 obese class II ≥35.0	Medical records	ADHD; other mental diseases: any mental health disorder(7-8y)	Mother’s age, race/ethnicity, education, insurance status, smoking, alcohol intake, marital status, and PCOS diagnosis; parental age difference and BMI; parental history of affective disorders; and child’s sex	7
Kong et al. (2020) [39]	Prospective study; Finland(2004); 34,892	Finland population-based registry cohort	Mother	WHO(NR); underweight <18.5 normal 18.50–24.99 overweight 25.00–29.99 obese class I 30.0–34.9 obese class II / III ≥35.0	Medical records	Other mental diseases: any mental health disorder(0-11y)	Offspring birth year, sex, perinatal problems and offspring birth weight according to gestational age, number of fetuses, mode of delivery, maternal age at delivery, parity, family situation, mother’s country of birth, and maternal smoking were obtained from the drugs and pregnancy database, maternal psychiatric disorders	8
Matias et al. (2021) [54]	Case–control study; USA(2003); 4,409	Study to Explore Early Development	Mother	NIH(2000) underweight <18.5 normal 18.50–24.99 overweight 25.00–29.99 obese class I 30.0–34.9 obese class II/ III≥35.0	Medical records	ASD; other mental diseases: DD without ASD(2-5y)	Maternal age, education, race/ethnicity, parity, smoking, income and site	7
Yim et al. (2021) [23]	Prospective study; USA(2001); 44,720	Nurses’ Mothers’ Cohort Study	Mother	WHO(2000); underweight <18.5 normal 18.50–24.99 overweight 25.00–29.99 obese ≥30.00	Self-report	ADHD;ASD(NR)	Grandmother (G0) race/ethnicity, grandmother and grandfather educational levels, grandfather occupation, grandmother lifetime history of depression, maternal (G1) year of birth, and G1 smoking status at baseline	7
Parker et al. (2022) [34]	Prospective study; USA(NA); 484	NR	Mother	CDC(2019); underweight <18.5 normal 18.50–24.99 overweight 25.00–29.99 obese ≥30.00	Self-report	Behavioral problems (5-12y)	Maternal age, maternal education, marital status, periconceptional alcohol use, periconceptional smoking, and weighted by stabilized inverse probabilities of participation	6

**Note:** ADHD, Attention-Deficit/Hyperactivity Disorder; ASD, Autism Spectrum Disorder; BMI, Body Mass Index; CDC, Centers for Disease Control and Prevention; DD, Development Delay; NIH, National Institutes of Health; IQ, Intelligence Quotient; NR, Not Report; PCOS, Polycystic Ovary Syndrome; PREOBE, a prospective observational cohort study (NCT01634464); USA, United States of America; WHO, World Health Organization.

### Risk of any mental illness

**Fig 2:**
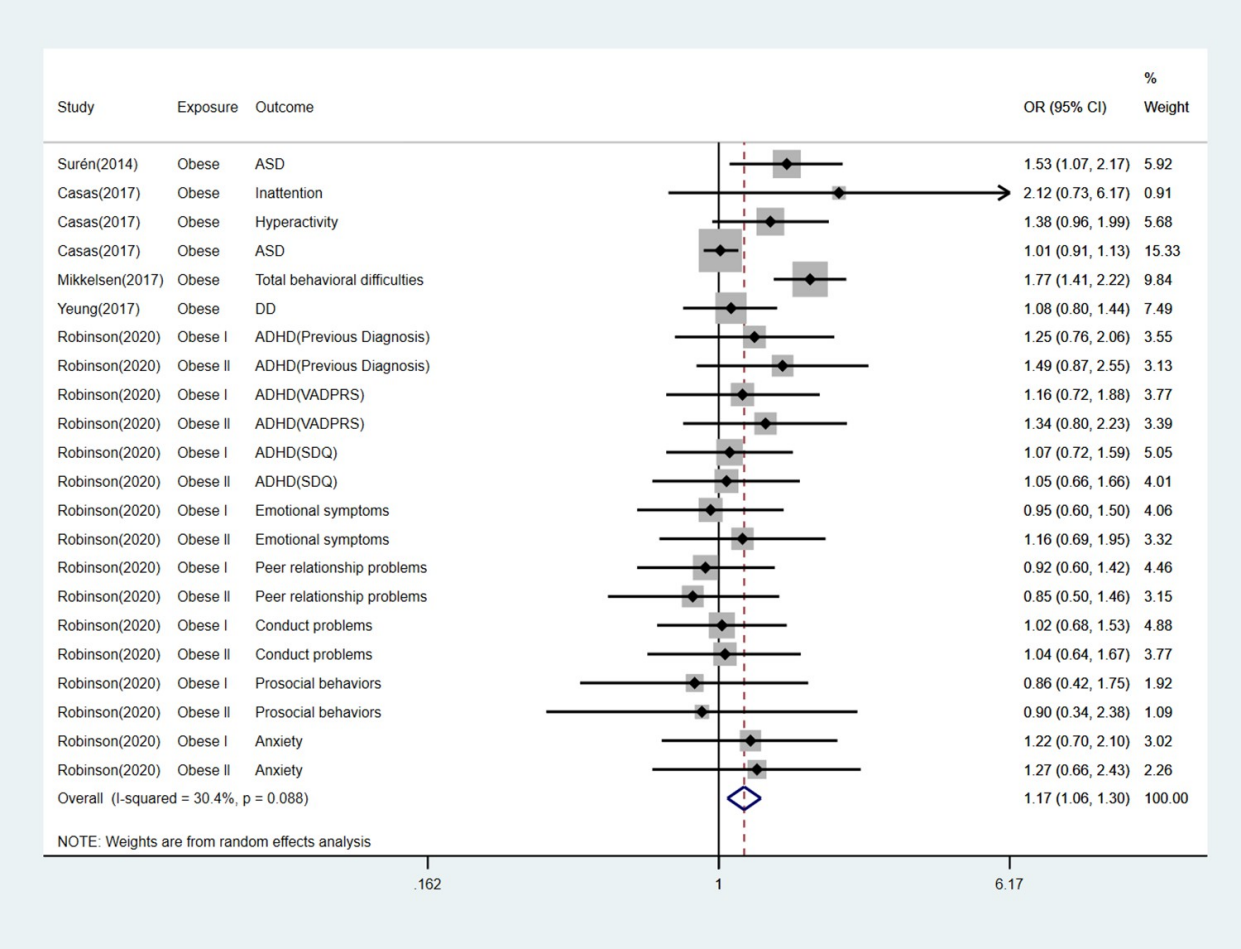
Forest plot for obese fathers and their offspring’s risk of any mental diseases.

We pooled the risk of any mental illness in offspring exposed to maternal and paternal overweight and obesity separately. For overweight mothers, there were 14% higher odds of having a child with any adverse mental outcome (OR, 1.14; 95% CI, 1.10–1.18; P < 0.001), For obese mothers, across 32 studies, there were 39% higher odds to have a child with any adverse mental outcome (OR, 1.39; 95% CI, 1.33–1.45; P < 0.001). As for paternal exposure, the pooled OR for paternal overweight was 1.03(95%CI, 0.95–1.11; P = 0.44), indicating that there was no significant association between paternal overweight and offspring mental illness. However, results across 5 studies indicated that fathers who were obese prior to pregnancy had 17% higher odds of having a child with any adverse neurodevelopmental outcome (OR, 1.17; 95% CI, 1.06–1.30; P < 0.001; Fig 2; Table 3).

**Table 3:** Summary of meta-analyses.

Subgroup	No. of studies	Overall effect		Heterogeneity	Publication bias	
		OR (95%)	P value	I^2^	Begg’s test	Egger’s test
**Maternal BMI weight group**						
Overweight	31	1.14(1.10,1.18)	<0.001	34.9%	0.20	0.17
Obesity	32	1.39(1.33, 1.45)	<0.001	57.3%	0.75	0.70
Overweight + Obesity	35	1.24(1.20, 1.28)	<0.001	71.8%	0.17	0.18
**Paternal BMI weight group**						
Overweight	4	1.03(0.95,1.11)	0.44	11.7%	0.71	0.02
Obesity	5	1.17(1.06,1.30)	0.003	30.4%	0.74	0.39
Overweight + Obesity	6	1.04(1.00, 1.09)	0.056	29.8%	0.23	0.39
**Maternal-Offspring mental diseases(Overweight)**						
ADHD	10	1.21(1.16,1.26)	<0.001	9.0%	0.50	0.92
ASD	13	1.09(1.04,1.15)	0.001	0%	0.30	0.50
Cognitive/intellectual delay	7	1.13(1.04, 1.24)	0.006	1.6%	0.09	0.26
Behavioral problems	9	1.28 (1.16, 1.41)	<0.001	10.8%	0.74	1.00
Other mental diseases	14	1.06(1.04,1.09)	<0.001	0%	0.77	0.62
**Maternal-Offspring mental diseases(Obesity)**						
ADHD	10	1.55(1.42,1.70)	<0.001	45.9%	0.70	0.45
ASD	15	1.37(1.22,1.55)	<0.001	60.8%	0.23	0.10
Cognitive/intellectual delay	7	1.40(1.21,1.63)	<0.001	60.0%	0.59	0.69
Behavioral problems	9	1.50(1.35, 1.66)	<0.001	12.8%	0.07	0.004
Other mental diseases	15	1.30(1.23,1.37)	<0.001	28.6%	0.88	0.68

**Note:** ADHD, Attention-Deficit/Hyperactivity Disorder; ASD, Autism Spectrum Disorder; BMI, Body Mass Index.

### Risk of specific mental illness

**Fig 3:**
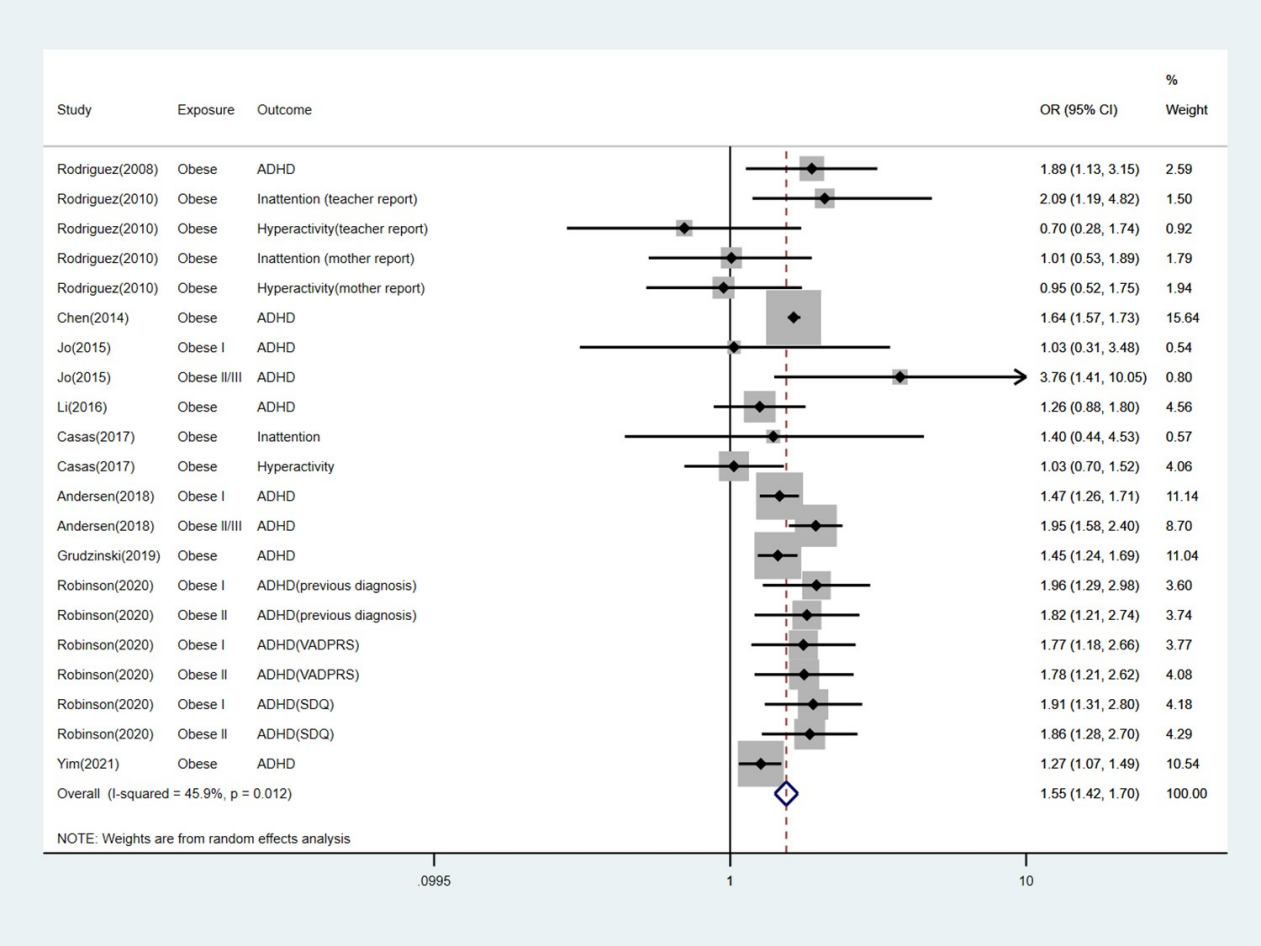
Forest plot for obese mothers and their offspring’s risk of ADHD.

**Fig 4:**
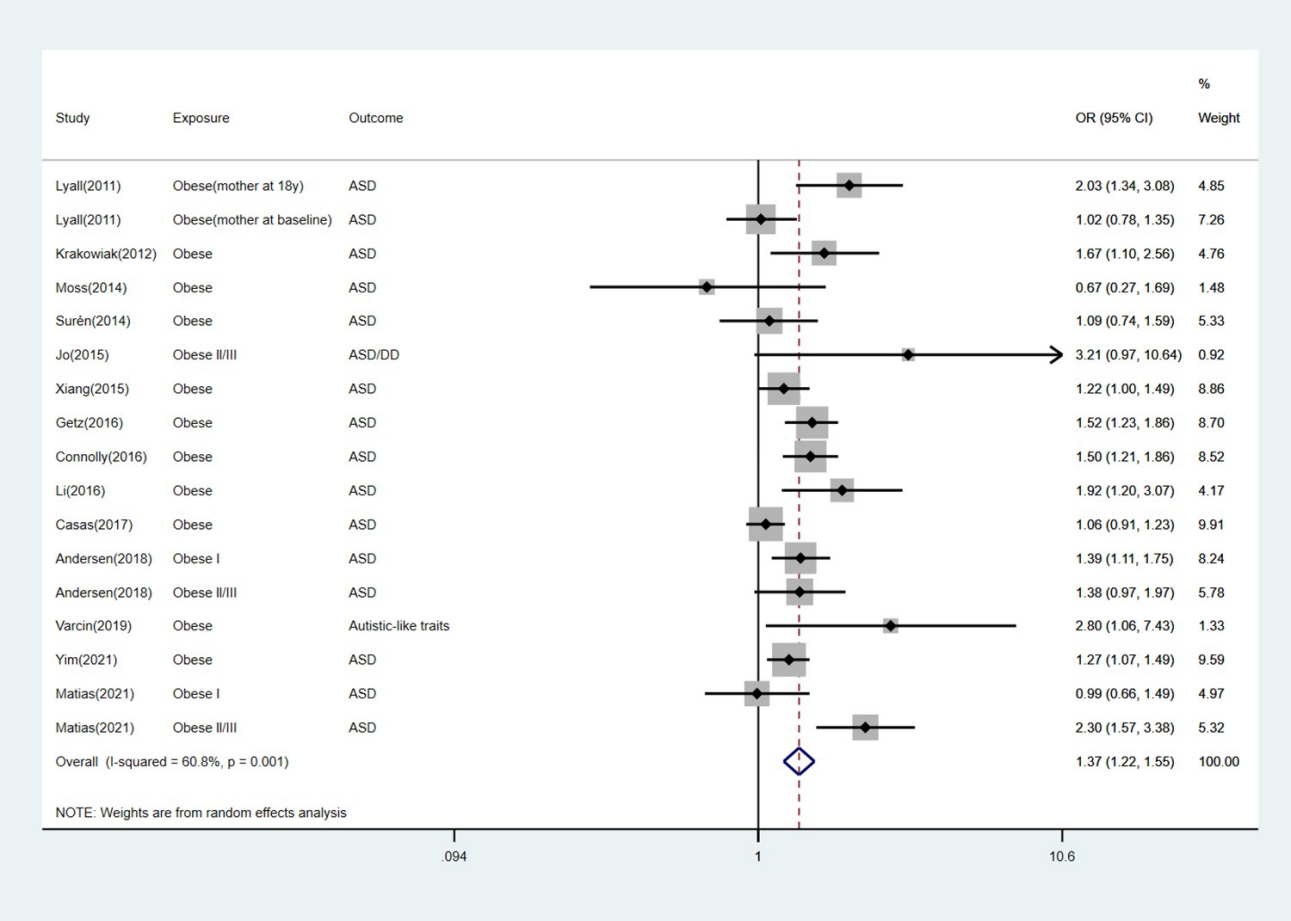
Forest plot for obese mothers and their offspring’s risk of ASD.

**Fig 5:**
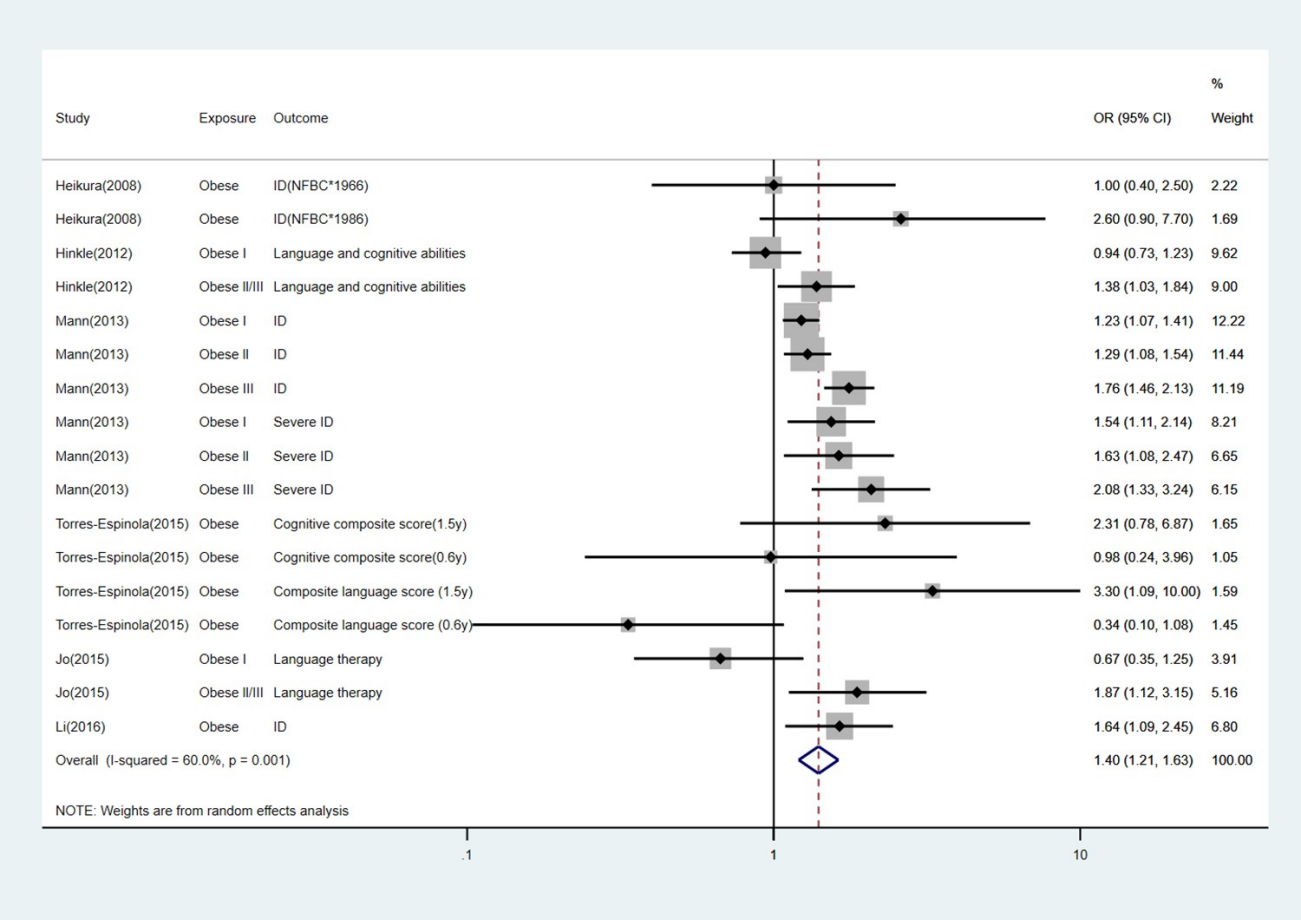
Forest plot for obese mothers and their offspring’s risk of cognitive/intellectual delay.

**Fig 6:**
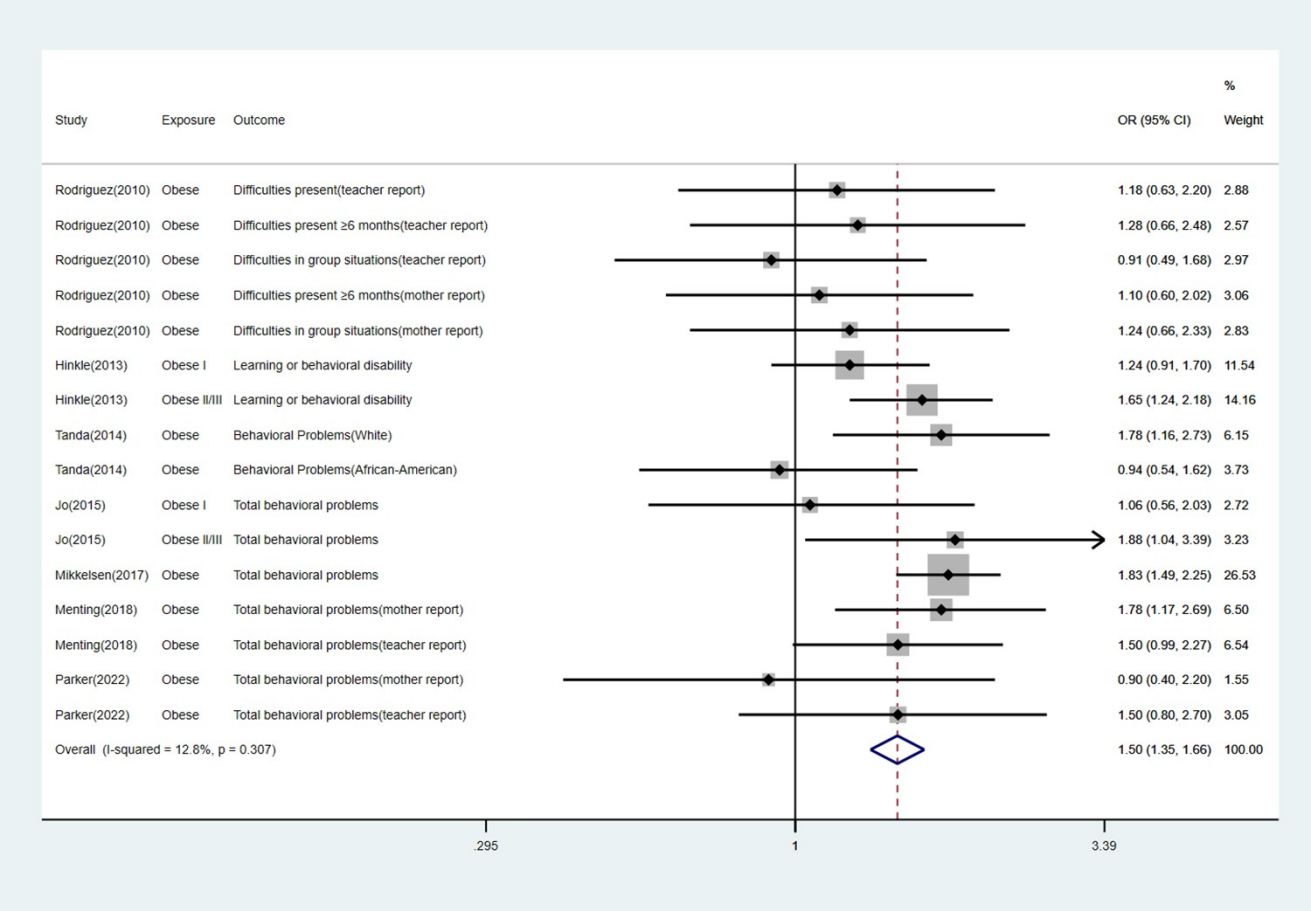
Forest plot for obese mothers and their offspring’s risk of behavioral problems.

**Fig 7:**
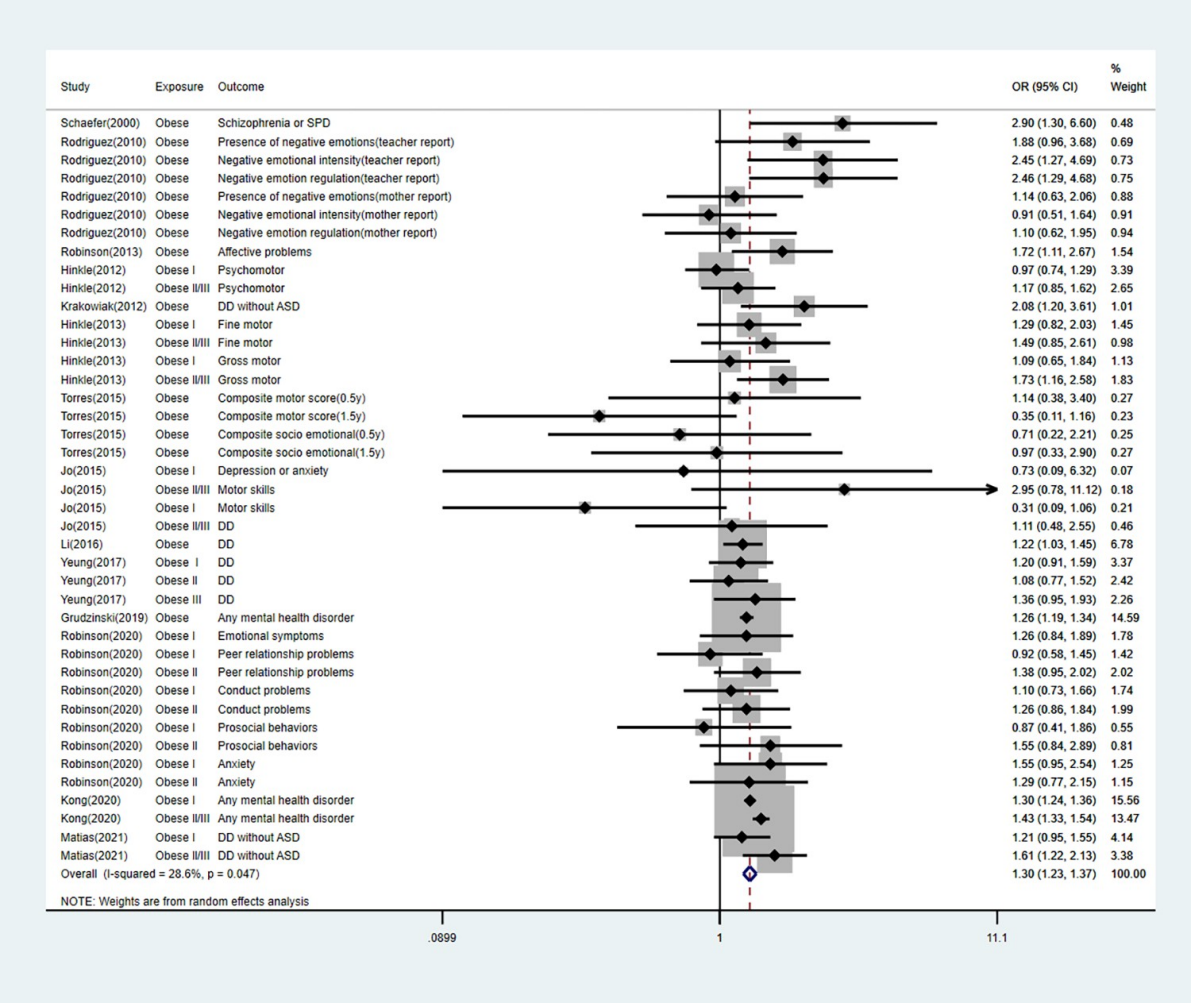
Forest plot for obese mothers and their offspring’s risk of other mental diseases.

We performed subgroup analyses according to specific offspring outcomes. The detailed information can be found in Table 3 and Figs 3–7.

### Publication bias

There was no evidence of publication bias according to Begg’s test and Egger’s test (all P>0.05) except the pooled analysis which show inconclusive evidence for an association between paternal overweight and offspring mental illnesses.

### Sensitivity analysis

Sensitivity analysis showed the results of the main analyses are robust. Studies with cohort study design produced an overall OR that was lower than those with case-control design. Slightly attenuated OR was observed for those studies ranked as having a lower quality score than those ranked as having a higher quality score (Table 4). We also investigated the influence of a single study on the overall risk estimate by performing leave-one-out analysis. The combined ORs of overall risk estimates were consistent and without apparent fluctuation (S3 Table).

**Table 4:** Sensitivity analysis of the effects of maternal pre-pregnancy overweight and obesity on offspring mental health.

	No. of studies	Overall effect(95%CI)	P	I^2^
**Study design**				
Prospective	24	1.24(1.19,1.29)	<0.001	72.5%
Retrospective	6	1.22(1.14,1.30)	<0.001	67.4%
Case–Control	5	1.30(1.13,1.48)	<0.001	68.8%
**Quality grade of study**				
High (NOS≥6)	30	1.24(1.20,1.28)	<0.001	73.1%
Low (NOS<6)	5	1.19(1.05,1.35)	0.008	19.9%

**Note:** NOS, Newcastle–Ottawa Quality Assessment Scale.

## Discussion

The findings of this meta-analysis are consistent with previous reviews that revealed a negative link between maternal pre-pregnancy overweight/obesity and the neurodevelopmental outcomes in offspring. Several inconsistencies should also be mentioned. First, to date, this is the first comprehensive meta-analysis not only investigated the effect of maternal overweight/obesity on offspring mental health but also fathers. Although far fewer studies have investigated paternal exposure than maternal, to some extent, we still conclude that paternal obesity has adverse effects on the neurodevelopment of their offspring. Second, for maternal weight exposure, we excluded studies that examined weight during pregnancy because of the neurological effects of gestational weight gain (GWG) on offspring. Evidence showed that whether GWG is insufficient or excessive may be important to fetal neurodevelopment [58–60]. However, the degree to which GWG and pre-pregnancy obesity independently or synergistically relate to an increased risk of offspring’s mental illnesses is not clear [61, 62]. Third, because we adhere to strict definitions of study design, the estimates of effect sizes are more accurate than they would be with weaker study designs, including cross-sectional studies. Forth, the included studies come from heterogeneous samples, countries, measures and designs so the effect size in the main analysis showed appropriate representativeness.

Multiple mechanisms have been proposed to explain the impact of maternal obesity on the offspring neurodevelopment. To our knowledge, obesity during pregnancy changes neuroendocrine, metabolic and inflammatory status [4].The increased cytokines, oxidative stress, and circulating hormones disorder “mother–placenta–fetus” system [63, 64]and the composition of lactational milk composition [65], resulting in potential changes to child’s neurodevelopment which in turn influences behaviour, emotion and cognition [66]. In addition to neuroinflammation, altered neuronal plasticity(brain-derived neurotrophic factor, notch signaling genes, proliferation of neuronal progenitor cells, abnormal synaptic stability) [67], impaired reward circuitry(dopaminergic and serotonergic signaling) [68] and dysregulated brain metabolism (leptin, insulin and oxytocin) and likely contributors. The alterations in the gut microbiome’s composition are another mechanism that has recently received attention [69]. Microbial metabolites may affect neuroendocrine regulation and brain development via passing through the placenta, vertically transmitting and lactation [65]. However, the detailed dynamics remain poorly understood.

The mechanisms explaining how the father may affect the neurodevelopment of offspring are still under debate [70].Obesity causes a variety of symptoms in men, including changes in their gut microbiome, hormone levels, and sperm health, which can lead to abnormalities in fertilization and embryonic development [71–73]. Additionally, seminal plasma has a distinct microbiome that can be altered by a high-fat diet(HFD), which means it can indirectly affect the offspring’s neurodevelopment and later brain function by passing along information about the father’s eating habits and metabolic condition [74].

Moreover, maternal and paternal obesity predispose offspring to poor neurodevelopment by epigenetic molecular mechanism, which is studied intensively in the field of neurobiology in animals [63]. The underlying molecular mechanisms involve DNA methylation, post-translational modifications of chromatin and/or histone proteins, and small noncoding RNAs (sncRNAs) alterations, which change the expression of genes involved in neuroplasticity [3]. Among the classical epigenetic mechanisms, DNA methylation is an attractive target for research. It has been reported that offspring of obese females exhibited DNA hypomethylation in gene promoters including those encoding proteins involved in dopamine uptake, and serotonergic and opioid signaling [3, 75, 76] and may be associated with the decreased folic acid in obese pregnant women [67]. Zhou et al. report that paternal HFD results in cognitive impairments in the F1 potentially due to the increased methylation of the BDNF gene promoter transmitted by F0 spermatozoa [77]. The recent clinical research found a link between paternal BMI and altered DNA methylation in cord blood nucleated cells, indicating that there are environment-sensitive parts of the sperm epigenome that respond to food and may transmit an ’epigenomic map’ that impacts offspring development [78]. Furthermore, sncRNAs have been hypothesized as another pathway of epigenetic transfer, particularly through the paternal line. These sperm-derived RNAs appear to be sensitive to a diverse range of psychological and physiological conditions, including stress exposure and nutrition, and are able to transmit intergenerational information through the paternal lineage [70, 72, 73]. Obesity-caused hyperglycemia, insulin resistance, and a proinflammatory lipotoxic environment could be the immediate source of such epigenetic changes, resulting in developmental brain problems in offspring [3].

Here, we detail the limitations that might influence the combined results. First, lack of resources meant that we excluded papers that were not in English and studies with continuous variables. Besides, it was very possible that studies with bad outcomes were unlikely to be published. Second, the exposure and outcome factors might have resulted in disparate results. The parental weight groupings and diagnostic criteria of neurodevelopment were variable, which may affect the results and contribute to the heterogeneity across several risk factors. Third, all the identified studies came from observational settings and were therefore subjected to confounding bias. Forth, the limited number of studies investigated fathers (only 6 studies) might affect the combined results. Also, 91% included studies were conducted in the America (51%) and Europe (40%), which limit generalizability to other populations across the world. Future studies should be performed in more countries outside of America and Europe.

## Conclusion

We found that the most recent evidence indicates the detrimental connections between parental pre-pregnancy overweight/obesity and offspring mental health. To reduce the incidence of mental problems in children, it may be prudent to conduct measures targeted at avoiding overweight and obesity in both mother and father. However, given the constraints discussed above, more research employing a range of study designs such as sibling comparison, children-of-twins designs, and within-family Mendelian randomization would be helpful in identifying the magnitude of the overall impact size. Also, the recent study showed grandmother underweight prior to pregnancy is associated with an increased risk of ADHD among grandchildren independent of maternal pre-pregnancy weight status, future work should focus on discovering mechanisms linking weight status and offspring mental health across generations.

## Supporting information

S1 Checklist PRISMA 2020 checklist. (DOCX)

S1 Table List of terms used for database searches. (DOCX)

S2 Table Neurodevelopmental outcomes. (DOCX)

S3 Table Sensitivity analyses were performed by removing results one by one. (DOCX)

S4 Table The corresponding plots of the leave-one-out analysis. (DOCX)

S5 Table The corresponding funnel plots of meta-analysis. (DOCX)

S6 Table Forest plot for sensitivity analysis. (DOCX)

S1 Appendix Original data extraction form. (XLSX)

S2 Appendix Sensitivity analysis data. (XLSX)
