# Supplementary material for: Effects of parental overweight and obesity on offspring’s mental health: A meta-analysis of observational studies
Source: PLoS One. 2022 Dec 22;17(12):e0276469. doi: 10.1371/journal.pone.0276469 (PMC9778529; doi:10.1371/journal.pone.0276469)
Supplement: S1 Table — (DOCX) [file pone.0276469.s002.docx]

**S1 Table. List of terms used for database searches**

| **Database**  **(n)** | **1. Parent** | **2.Obesity** | **3.Offspring** | **4.Mental disorder** | **5. Study type** | **6.Combination** |
| --- | --- | --- | --- | --- | --- | --- |
| **Pubmed**  **(1,125)**  **Cochrane**  **(3,378)** | "Parents"[Mesh] OR "Single Parent"[Mesh] OR "Parent-Child Relations"[Mesh] OR "Mothers"[Mesh] OR "Maternal Exposure"[Mesh] OR "Mother-Child Relations"[Mesh] OR "Maternal-Fetal Relations"[Mesh] OR "Fathers"[Mesh] OR "Paternal Exposure"[Mesh] OR "Father-Child Relations"[Mesh] OR (parent*) OR (maternal) OR (mother*) OR (paternal) OR (father*) | "Body Mass Index"[Mesh] OR "Skinfold Thickness"[Mesh] OR "Waist Circumference"[Mesh] OR "Obesity"[Mesh] OR "Overweight"[Mesh] OR "Adiposity"[Mesh] OR "Waist-Hip Ratio"[Mesh] OR "Waist-Height Ratio"[Mesh] OR "Electric Impedance"[Mesh] OR (BMI) OR ("body weight") OR (obes*) OR ("over weight") OR (adipos*) OR ("metabolic syndrome") OR (skinfold) OR ("skin fold") OR ("waist-to-hip ratio") OR (fat) OR ("fat mass index") OR ("fat free mass index") OR ("body adiposity index") OR ("hip circumference") OR ("neck circumference") OR ("waist-to-height ratio") OR ("bioelectric impedance") OR ("electric resistance") OR ("near infrared interactance") OR ("benn index") OR ("rohrer index") OR ("ponderal index") OR ("corpulence index") OR ("sagittal abdominal diameter") | "Child"[Mesh] OR "Infant"[Mesh] OR "Adolescent"[Mesh] OR (infant*) OR (pediatric*) OR (child*) OR (preschool) OR (schoolchild) OR ("school age") OR (schoolage) OR (adolescen*) OR (juvenile) OR (youth) OR (teen*) OR (youngster) OR (girl) OR (girls) OR (boy) OR (boys) OR (kid) OR (kids) OR (offspring) | "Mental Disorders"[Mesh] OR "Neurodevelopmental Disorders"[Mesh] OR "Neurocognitive Disorders"[Mesh] OR ("mental illness") OR ("psychiatric disorder") OR ("psychiatric illness") OR ("mental health") OR ("child mental disorder") OR ("organic mental disorders") OR ("substance use disorders") OR ("alcohol abuse")OR ("cannabis use") OR ("psychotic disorders") OR ("schizophrenia spectrum disorder") OR (schizo*) OR ("acute psychoses") OR ("mood disorders") OR ("bipolar disorder") OR (depress*) OR (anxiety) OR (angst) OR (nervous*) OR (neurot*) OR (anxiousness) OR ("stress disorders") OR (phobi*) OR (traumatic) OR ("eating disorders") OR ("anorexia nervosa") OR (bulimia) OR ("obsessive-compulsive disorder") OR (OCD) OR ("personality disorders") OR ("autism spectrum disorders") OR ("childhood autism") OR (ASD) OR ("asperger syndrome") OR ("intellectual disability") OR ("other developmental disorders") OR (ADHD) OR ("attention deficit hyperactivity disorder") OR (ODD) OR (CD) OR (MDD) OR ("oppositional defiant disorder") OR ("conduct disorder") OR ("attachment disorders") OR ("tic disorders") | "Case-Control Studies"[Mesh] OR "Cohort Studies"[Mesh] OR "Risk Factors"[Mesh] OR "Matched-Pair Analysis"[Mesh] OR "Control Groups"[Mesh] OR ("comparative study") OR ("control group") OR ("case control") OR ("case comparison") OR ("longitudinal studies") OR ("follow-up studies") OR ("prospective studies") OR ("retrospective studies") OR ("epidemiological studies") ("rate ratio") OR ("hazard ratio") OR ("relative risk") OR ("odds ratio") | 1 and 2 and 3 and 4 and 5 |
| **Embase**  **(11,371)** | 'parent'/exp OR 'single parent'/exp OR 'child parent relation'/exp OR 'mother'/exp OR 'maternal exposure'/exp OR 'mother child relation'/exp OR 'mother fetus relationship'/exp OR 'father'/exp OR 'paternal exposure'/exp OR 'father child relation'/exp OR parenting OR parental OR parent* OR maternal OR mother* OR paternal OR father* | 'body mass'/exp OR 'skinfold thickness'/exp OR 'waist circumference'/exp OR 'obesity'/exp OR 'waist hip ratio'/exp OR 'waist to height ratio'/exp OR 'impedance'/exp OR bmi OR 'body weight' OR obes* OR adipos* OR 'over weight' OR 'metabolic syndrome' OR skinfold OR 'skin fold' OR 'waist-to-hip ratio' OR fat OR 'fat mass index' OR 'fat free mass index' OR 'body adiposity index' OR 'hip circumference' OR 'neck circumference' OR 'waist-to-height ratio' OR 'bioelectric impedance' OR 'electric resistance' OR 'near infrared interactance' OR 'benn index' OR 'rohrer index' OR 'ponderal index' OR 'corpulence index' OR 'sagittal abdominal diameter' | 'child'/exp OR 'infant'/exp OR 'adolescent'/exp OR pediatric OR infant* OR pediatric* OR child* OR preschool OR schoolchild OR 'school age' OR schoolage OR adolescen* OR juvenile OR youth OR teen* OR youngster OR girl OR girls OR boy OR boys OR kid OR kids OR teen OR offspring | 'mental disease'/exp OR 'disorders of higher cerebral function'/exp OR 'mental illness' OR 'psychiatric disorder' OR 'psychiatric illness' OR 'mental health' OR 'child mental disorder' OR 'organic mental disorders' OR 'substance use disorders' OR 'alcohol abuse' OR 'cannabis use' OR 'psychotic disorders' OR 'schizophrenia spectrum disorder' OR schizo* OR 'acute psychoses' OR 'mood disorders' OR 'bipolar disorder' OR 'depressive episode' OR depress* OR anxiety OR angst OR nervous* OR neurot* OR anxiousness OR 'stress disorders' OR phobi* OR traumatic OR 'eating disorders' OR 'anorexia nervosa' OR bulimia OR 'obsessive-compulsive disorder' OR ocd OR 'personality disorders' OR 'autism spectrum disorders' OR 'childhood autism' OR asd OR 'asperger syndrome' OR 'intellectual disability' OR 'other developmental disorders' OR adhd OR 'attention deficit hyperactivity disorder' OR odd OR cd OR mdd OR 'oppositional defiant disorder' OR 'conduct disorder' OR 'attachment disorders' OR 'tic disorders' | 'case control study'/exp OR 'cohort analysis'/exp OR 'risk factor'/exp OR 'control group'/exp OR 'clinical article'/exp OR 'controlled study'/exp OR 'major clinical study'/exp OR 'prospective study'/exp OR 'comparative study' OR 'control group' OR 'case control' OR 'case comparison' OR 'longitudinal studies' OR 'follow-up studies' OR 'prospective studies' OR 'retrospective studies' OR 'epidemiological studies' OR 'rate ratio' OR 'hazard ratio' OR 'relative risk' OR 'odds ratio' | 1 and 2 and 3 and 4 and 5 |
| **PsyINFO**  **(3,046)**  **Web of science**  **(3,591)** | "Parents" OR "Single Parent" OR "Parent-Child Relations" OR "Mothers" OR "Maternal Exposure" OR "Mother-Child Relations"OR "Maternal-Fetal Relations" OR "Fathers" OR "Paternal Exposure"OR "Father-Child Relations" OR (parent*) OR (maternal) OR (mother*) OR (paternal) OR (father*) | "Body Mass Index" OR "Skinfold Thickness" OR "Waist Circumference" OR "Obesity" OR "Overweight" OR "Adiposity" OR "Waist-Hip Ratio" OR "Waist-Height Ratio"OR "Electric Impedance" OR (BMI) OR ("body weight") OR (obes*) OR ("over weight") OR (adipos*) OR ("metabolic syndrome") OR (skinfold) OR ("skin fold") OR ("waist-to-hip ratio") OR (fat) OR ("fat mass index") OR ("fat free mass index") OR ("body adiposity index") OR ("hip circumference") OR ("neck circumference") OR ("waist-to-height ratio") OR ("bioelectric impedance") OR ("electric resistance") OR ("near infrared interactance") OR ("benn index") OR ("rohrer index") OR ("ponderal index") OR ("corpulence index") OR ("sagittal abdominal diameter") | "Child" OR "Infant" OR "Adolescent" OR (infant*) OR (pediatric*) OR (child*) OR (preschool) OR (schoolchild) OR ("school age") OR (schoolage) OR (adolescen*) OR (juvenile) OR (youth) OR (teen*) OR (youngster) OR (girl) OR (girls) OR (boy) OR (boys) OR (kid) OR (kids) OR (offspring) | "Mental Disorders" OR "Neurodevelopmental Disorders" OR "Neurocognitive Disorders" OR ("mental illness") OR ("psychiatric disorder") OR ("psychiatric illness") OR ("mental health") OR ("child mental disorder") OR ("organic mental disorders") OR ("substance use disorders") OR ("alcohol abuse")OR ("cannabis use") OR ("psychotic disorders") OR ("schizophrenia spectrum disorder") OR (schizo*) OR ("acute psychoses") OR ("mood disorders") OR ("bipolar disorder") OR (depress*) OR (anxiety) OR (angst) OR (nervous*) OR (neurot*) OR (anxiousness) OR ("stress disorders") OR (phobi*) OR (traumatic) OR ("eating disorders") OR ("anorexia nervosa") OR (bulimia) OR ("obsessive-compulsive disorder") OR (OCD) OR ("personality disorders") OR ("autism spectrum disorders") OR ("childhood autism") OR (ASD) OR ("asperger syndrome") OR ("intellectual disability") OR ("other developmental disorders") OR (ADHD) OR ("attention deficit hyperactivity disorder") OR (ODD) OR (CD) OR (MDD) OR ("oppositional defiant disorder") OR ("conduct disorder") OR ("attachment disorders") OR ("tic disorders") OR (behav*) | "Case-Control Studies" OR "Cohort Studies"OR "Risk Factors"OR "Matched-Pair Analysis" OR "Control Groups" OR ("comparative study") OR ("control group") OR ("case control") OR ("case comparison") OR ("longitudinal studies") OR ("follow-up studies") OR ("prospective studies") OR ("retrospective studies") OR ("epidemiological studies") ("rate ratio") OR ("hazard ratio") OR ("relative risk") OR ("odds ratio") | 1 and 2 and 3 and 4 and 5 |

* Boolean search modifier used for root word/stem search.
