# Supplementary material for: Effects of parental overweight and obesity on offspring’s mental health: A meta-analysis of observational studies
Source: PLoS One. 2022 Dec 22;17(12):e0276469. doi: 10.1371/journal.pone.0276469 (PMC9778529; doi:10.1371/journal.pone.0276469)
Supplement: S2 Table — (DOCX) [file pone.0276469.s003.docx]

**S2 Table. Neurodevelopmental outcomes**

| **Outcomes** | **Study** | **Diagnostic method** | **Outcome definition** | **Outcome information** |
| --- | --- | --- | --- | --- |
| **ADHD** | Rodriguez et al., 2008 | SDQ; RB2 | A total of 4 points or more | Teacher report |
|  | Rodriguez, 2010 | DSM-IV | NR | Teacher report |
|  | Chen et al., 2014 | ICD-9; ICD-10; DSM-IV-TR | One primary diagnosis and up to eight secondary diagnoses | Medical record |
|  | Jo et al., 2015 | Previous Diagnosis | NR | Mother report |
|  | Li et al., 2016 | ICD-9 | Code 314.0–314.9 | Medical record |
|  | Casas et al., 2017 | DSM-IV | Cronbach’s alpha coefficient obtaining was 0.92 (inattention) and 0.90 (hyperactivity) | Teacher report |
|  | Andersen et al., 2018 | ICD-10 | Code F90.0/F90.1/F98.8 or received at least one prescription for ADHD medication | Medical record |
|  | Grudzinski et al., 2019 | ICD-9 | Code 314 | Medical record |
|  | Robinson et al., 2020 | Previous Diagnosis;  VADPRS; SDQ | NR; any of the 8 performance items and often or very often on 6 items for the subtype scales; hyperactivity/inattention ≥5 | Mother report |
|  | Yim et al., 2021 | Previous Diagnosis | NR | Mother report |
| **ASD** | Lyall et al., 2011 | Previous Diagnosis | NR | Mother report |
|  | Krakowiak et al., 2012 | ADI-R and ADOS | Children with scores above the ASD cutoff (≥15) were assessed with the ADOS and ADI-R, and reclassified to ASD if criteria were satisfied | Researcher report |
|  | Moss & Chugani, 2014 | Previous Diagnosis | NR | Parent report |
|  | Surén et al., 2014 | DSM; ICD-10 | Codes 299.00 and 299.80;F84.0,F84.1,F84.5,F84.8,F84.9 | Medical records |
|  | Jo et al., 2015 | Previous Diagnosis | NR | Mother report |
|  | Xiang et al., 2015 | ICD-9 | Codes 299.x or equivalent KPSC hospitals codes | Medical records |
|  | Getz et al., 2016 | Previous Diagnosis | NR | Medical records |
|  | Connolly et al., 2016 | ICD-9 | Codes 299.00 | Medical records |
|  | Li et al., 2016 | ICD-9 | Codes 299.x | Medical records |
|  | Casas et al., 2017 | CAST | Cronbach’s alpha coefficient obtaining was 0.64 | Psychologists report |
|  | Andersen et al., 2018 | ICD-10 | Code F84.0,F84.1,F84.5,F84.8,F84.9 | Medical records |
|  | Shen et al., 2018 | DSM-IV-TR | NR | Psychologists report |
|  | Varcin et al., 2019 | AQ | Total score higher than 26 | Self-report |
|  | Yim et al., 2021 | Previous Diagnosis | NR | Mother report |
|  | Matias et al., 2021 | Previous Diagnosis; SCQ | NR; SCQ score ≥11, and/or ASD symptoms noted by a SEED research clinician | Parent report |
| **Cognitive/intellectual delay** | Heikura et al., 2008 | ICD-9 | The definition of ID as an IQ of 70 or below | Psychologists report |
|  | Brion et al., 2011 | WISC-II; DANVA; MTCQ | NR | Psychologists report |
|  | Hinkle et al., 2012 | BSF-R; MDI | Researcher used a cutoff of score ＜40 to represent children with mild or severe delay | Researcher report |
|  | Mann et al.2013 | ICD-9 | Code 317-319 | Medical records |
|  | Torres-Espinola et al., 2015 | BSID-III | Bayley scores were all dichotomized using the 50th percentile | Psychologists report |
|  | Jo et al., 2015 | Previous Diagnosis | NR | Mother report |
|  | Li et al., 2016 | ICD-9 | Code 317, 318.0–318.2, and 319 | Medical record |
| **Behavioral problems** | Brion et al., 2011 | SDQ; CBCL | NR | Teacher and mother report |
|  | Rodriguez, 2010 | SDQ | NR | Teacher report |
|  | Hinkle et al., 2013 | BSID-II | NR | Mother report |
|  | Tanda & Salsberry, 2014 | BPI | Higher than population-normed scores | Mother report |
|  | Antoniou et al., 2014 | CBCL | Children were categorized as being in the borderline/clinical range when their T score ≥60. | Mother report |
|  | Jo et al., 2015 | SDQ | The 4 SDQ subscale scores and the total difficulties score were dichotomized as low versus moderate/high difficulties. | Mother report |
|  | Mikkelsen et al., 2017 | SDQ | Total difficulties score (girls: 12–40 and boys 14–40) | Parent report |
|  | Menting et al., 2018 | SDQ | Dichotomized into normal and borderline/clinical score using the 90th percentile score | Teacher and mother report |
|  | Parker et al., 2022 | CBCL | Broadband scales: T ≥60; syndrome scales T ≥65 | Teacher and mother report |
| **Other mental diseases** | Schaefer et al., 2000 | Diagnostic interview | NR | Psychologists report and medical record |
|  | Rodriguez, 2010 | SDQ | NR | Teacher and mother report |
|  | Robinson, 2013 | CBCL; DSM-IV | Researcher applied the recommended clinical cutoff scores (by age and sex) to the T-scores to obtain a binary variable indicative of clinically significant affective problems. | Caregiver report |
|  | Hinkle et al., 2012 | BSF-R; PDI | Researcher used a cutoff of score ＜40 to represent children with mild or severe delay | Researcher report |
|  | Krakowiak et al., 2012 | MSEL; VABS | Children with composite scores ,＜70 on the MSEL and/or VABS | Researcher report |
|  | Hinkle et al., 2013 | BSID-II | Low fine motor function: children did not pass the gate building assessment and at least half of the copy shape assessments; low gross motor function: passed less than half of the gross motor tasks were classified | Researcher report |
|  | Torres-Espinola et al., 2015 | BSID-III | Bayley scores were all dichotomized using the 50th percentile | Psychologists report |
|  | Jo et al., 2015 | Previous Diagnosis | NR | Mother report |
|  | Li et al., 2016 | ICD-9 | Code 315.0–315.5 | Medical record |
|  | Yeung et al., 2017 | ASQ | Scores 2 SDs below the mean for the child’s age per ASQ instructions | Parent report |
|  | Grudzinski et al., 2019 | ICD-9 | Code 296, 300, 308, 309, 311-314 | Medical record |
|  | Robinson et al., 2020 | SDQ | Emotional symptoms ≥3; peer relationship problems ≥2; conduct problems≥2; prosocial behaviors ≤6; anxiety Previous Diagnosis | Mother report |
|  | Kong et al., 2020 | ICD-10 | F30-F39, F40-F43, F50-F51, F60-F69, F70-F79, F80-F84, F90-F93, F98 | Medical record |
|  | Matias et al., 2021 | Previous diagnosis; SCQ | NR; SCQ score ≥11 | Parent report |

Abbreviations: ADHD, Attention-Deficit/Hyperactivity Disorder; ADI-R, Autism Diagnostic Interview, Revised; ADOS, Autism Diagnostic Observation Schedule; AQ, Autism Spectrum Quotient; ASD, Autism Spectrum Disorder; ASQ, Ages and Stages Questionnaire; BPI, Behavior Problem Index; BSF-R, Bayley Short Form--Research Edition; BSID-II, Bayley Scales of Infant Development, Second Edition; BSID-III, Bayley Scales of Infant Development, Third Edition; CAST, Childhood Asperger Syndrome Test; CBCL, Child Behavior Checklist; DANVA, Diagnostic Analysis of Nonverbal Accuracy; DSM, Diagnostic and Statistical Manual of Mental Disorders; DSM-IV, Diagnostic and Statistical Manual of Mental Disorders, Forth Edition; DSM-IV-TR ,Diagnostic and Statistical Manual of Mental Disorders, Fourth Edition, Text Revision; ICD-9, International Classification of Diseases Ninth Revision; ICD-10, International Classification of Diseases, Tenth Revision; ID, Intellectual Disability; IQ, Intelligence Quotient; KPSC, Kaiser Permanente Southern California; MDI, Mental Development Index; MSEL, Mullen Scales of Early Learning; MTCQ, MacArthur Toddler Communication Questionnaire; NR, Not Reported; PDI, Psychomotor Development Index; RB2, Rutter’s Behavior scale(B2); SCQ ,Social Communication Questionnaire; SD, Standard Deviation ;SDQ, Strength and Difficulties Questionnaire; SEED, Study to Explore Early Development; VABS, Vineland Adaptive Behavior Scales; VADPRS, Vanderbilt ADHD Diagnostic Parent Rating Scale; WISC-II, Wechsler Intelligence Scale for Children, Second Edition
