# Supplementary material for: Effects of parental overweight and obesity on offspring’s mental health: A meta-analysis of observational studies
Source: PLoS One. 2022 Dec 22;17(12):e0276469. doi: 10.1371/journal.pone.0276469 (PMC9778529; doi:10.1371/journal.pone.0276469)
Supplement: S3 Table — (DOCX) [file pone.0276469.s004.docx]

**S3 Table. Sensitivity analyses performed by removing results one by one**

| **Subgroup** | **Combined results range** | |
| --- | --- | --- |
|  | **Lower OR(95%CI)** | **Upper OR(95%CI)** |
| **Maternal BMI weight group** | | |
| Overweight | 1.13(1.09,1.16) | 1.15(1.11, 1.18) |
| Obesity | 1.38(1.32,1.45) | 1.39(1.33,1.46) |
| Overweight + Obesity | 1.23(1.19,1.27) | 1.24(1.20,1.28) |
| **Paternal BMI weight group** | | |
| Overweight | 1.00(0.91,1.10) | 1.06(0.99,1.13) |
| Obesity | 1.08(1.00,1.16) | 1.22(1.10, 1.35) |
| Overweight + Obesity | 1.03(0.99,1.06) | 1.05(1.00,1.10) |
