# Supplementary material for: Effects of parental overweight and obesity on offspring’s mental health: A meta-analysis of observational studies
Source: PLoS One. 2022 Dec 22;17(12):e0276469. doi: 10.1371/journal.pone.0276469 (PMC9778529; doi:10.1371/journal.pone.0276469)
Supplement: S4 Table — (DOCX) [file pone.0276469.s005.docx]

**S4 Table. The corresponding plots of the leave-one-out analysis**

| **Subgroup** | **Figure** |
| --- | --- |
| **Maternal BMI weight group** | |
| Overweight | **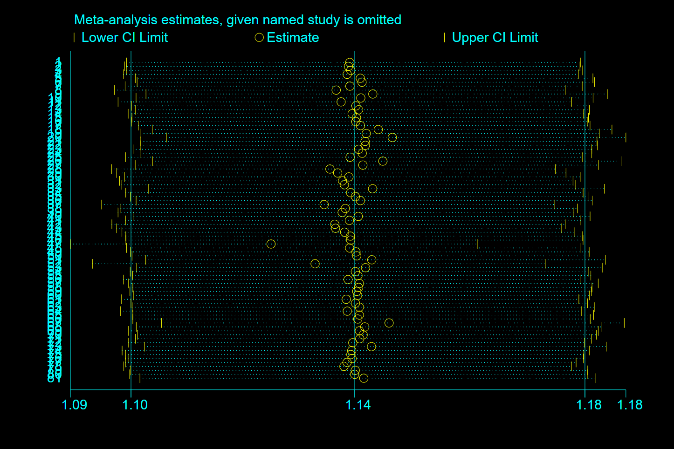** |
| Obesity | **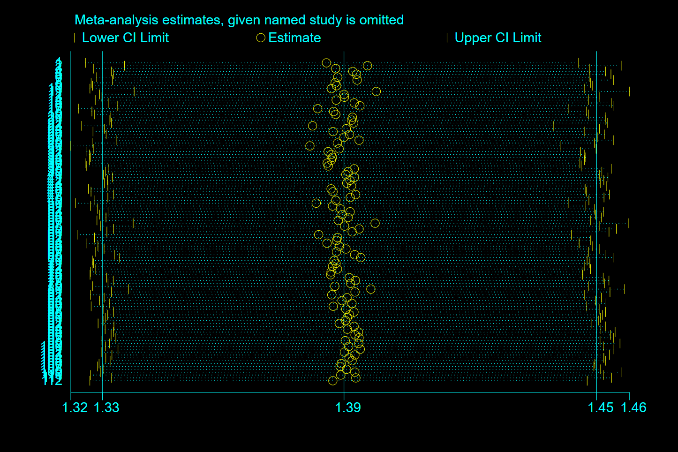** |
| Overweight + Obesity | **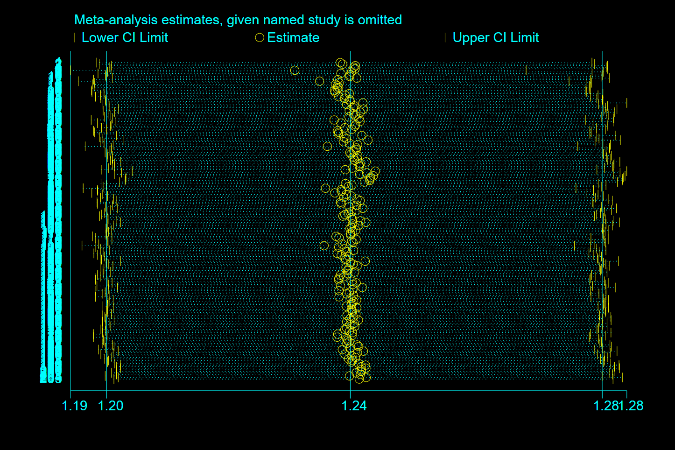** |
| **Paternal BMI weight group** |  |
| Overweight | **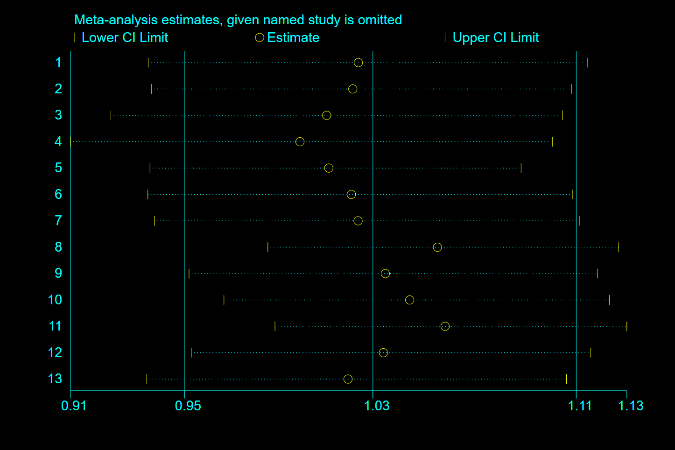** |
| Obesity | **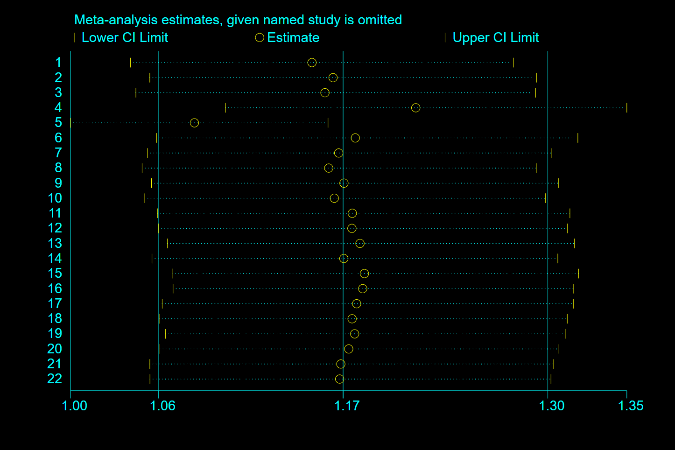** |
| Overweight + Obesity | **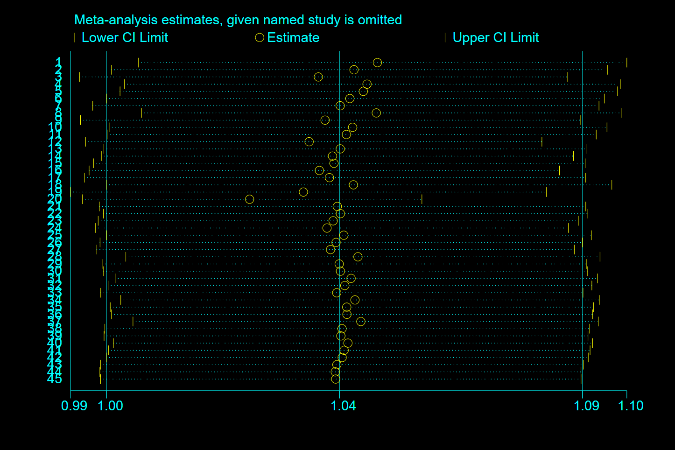** |
