# Supplementary material for: Effects of parental overweight and obesity on offspring’s mental health: A meta-analysis of observational studies
Source: PLoS One. 2022 Dec 22;17(12):e0276469. doi: 10.1371/journal.pone.0276469 (PMC9778529; doi:10.1371/journal.pone.0276469)
Supplement: S5 Table — (DOCX) [file pone.0276469.s006.docx]

**S5 Table. The corresponding funnel plots of meta-analysis**

| **Subgroup** | **Figure** |
| --- | --- |
| **Maternal BMI weight group** |  |
| Overweight | **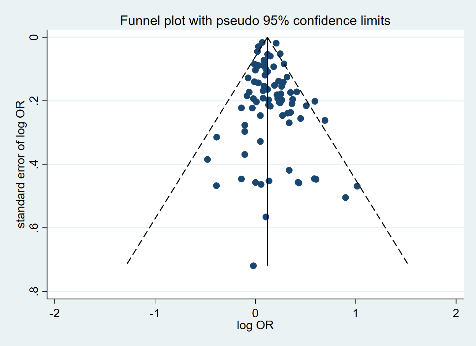** |
| Obesity | **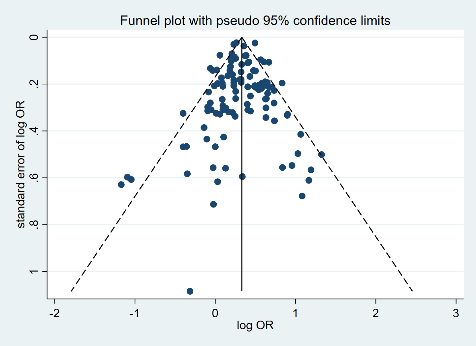** |
| Overweight + Obesity | **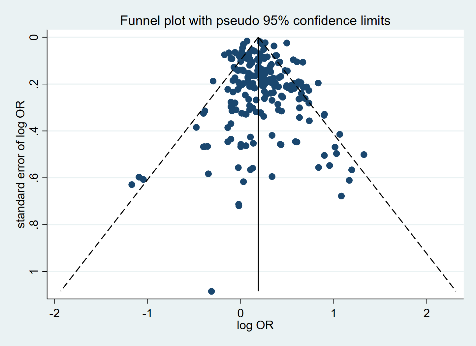** |
| **Paternal BMI weight group** |  |
| Overweight | **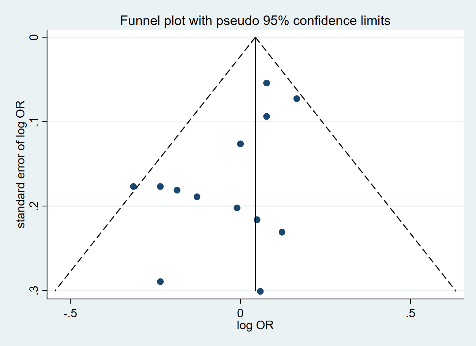** |
| Obesity | **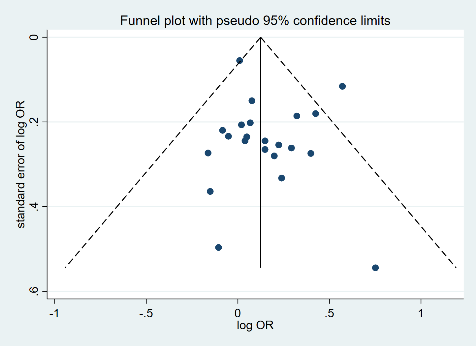** |
| Overweight + Obesity | **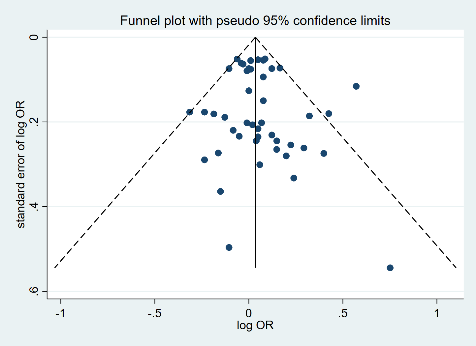** |
| **Maternal-Offspring mental diseases（Overweight）** |  |
| ADHD | **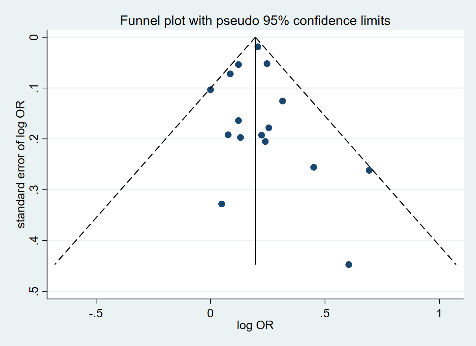** |
| ASD | **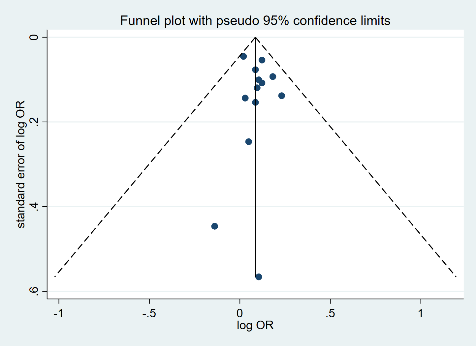** |
| Cognitive/intellectual delay | **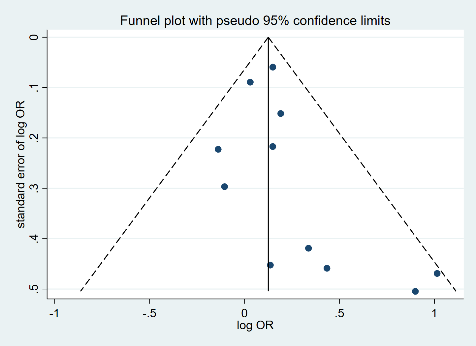** |
| Behavioral problems | **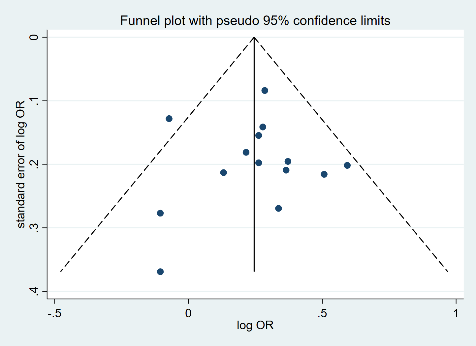** |
| Other mental diseases | **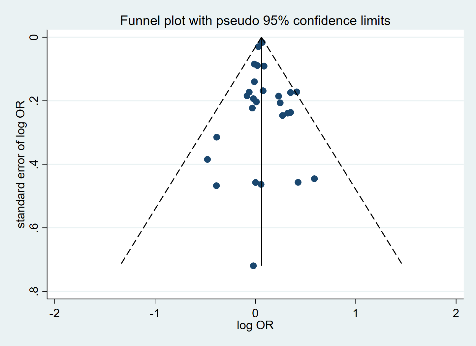** |
| **Maternal-Offspring mental diseases（Obesity）** |  |
| ADHD | **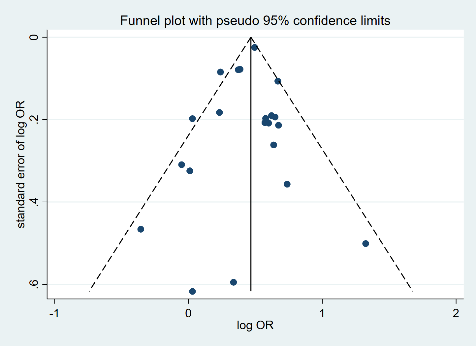** |
| ASD | **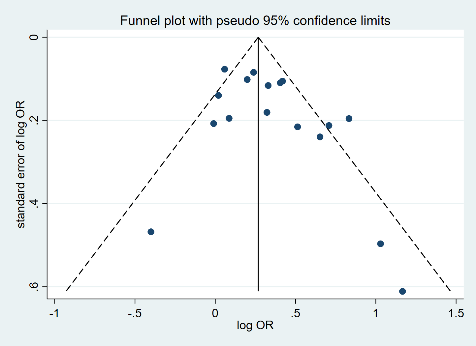** |
| Cognitive/intellectual delay | **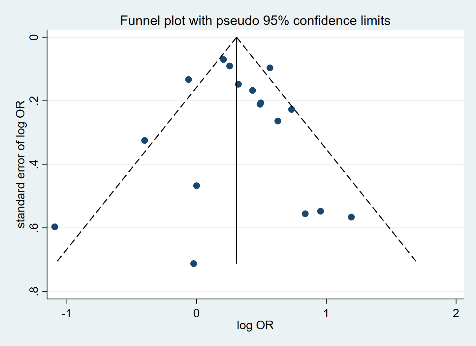** |
| Behavioral problems | **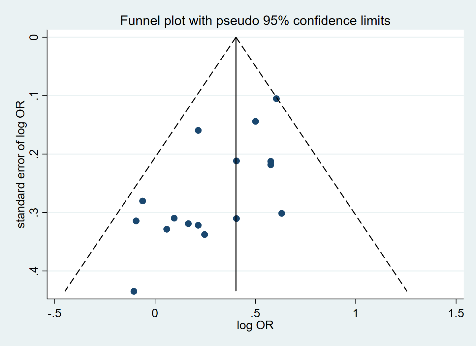** |
| Other mental diseases | **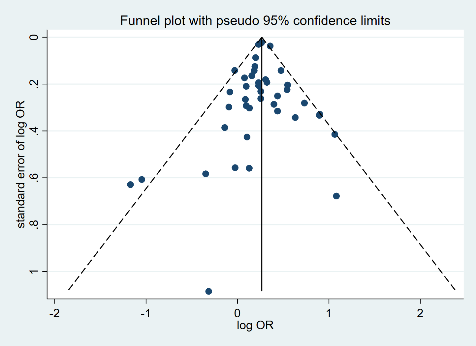** |
