# Supplementary material for: Effects of parental overweight and obesity on offspring’s mental health: A meta-analysis of observational studies
Source: PLoS One. 2022 Dec 22;17(12):e0276469. doi: 10.1371/journal.pone.0276469 (PMC9778529; doi:10.1371/journal.pone.0276469)
Supplement: S6 Table — (DOCX) [file pone.0276469.s007.docx]

**S6 Table. Forest plot for Sensitivity analysis.**

|  | **Figure** |
| --- | --- |
| **Study design** |  |
| Prospective | **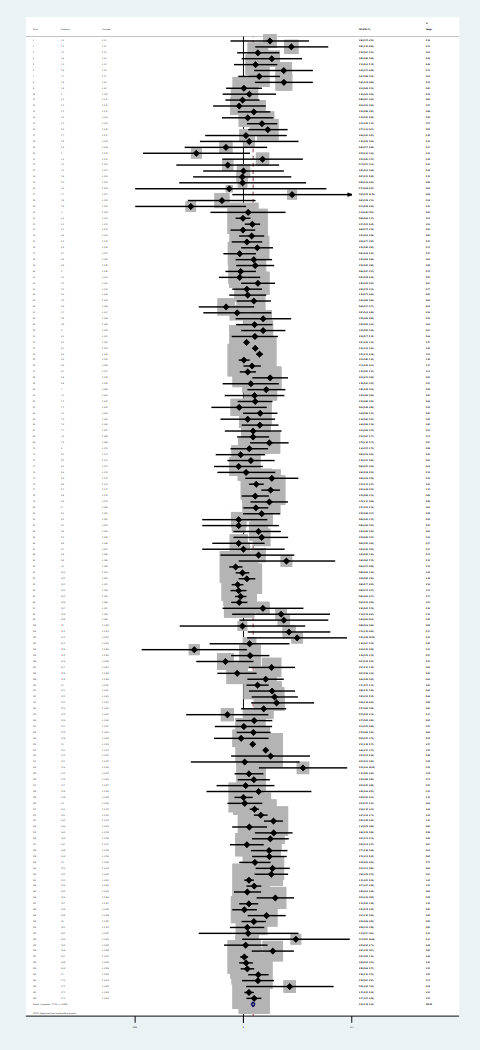** |
| Retrospective | **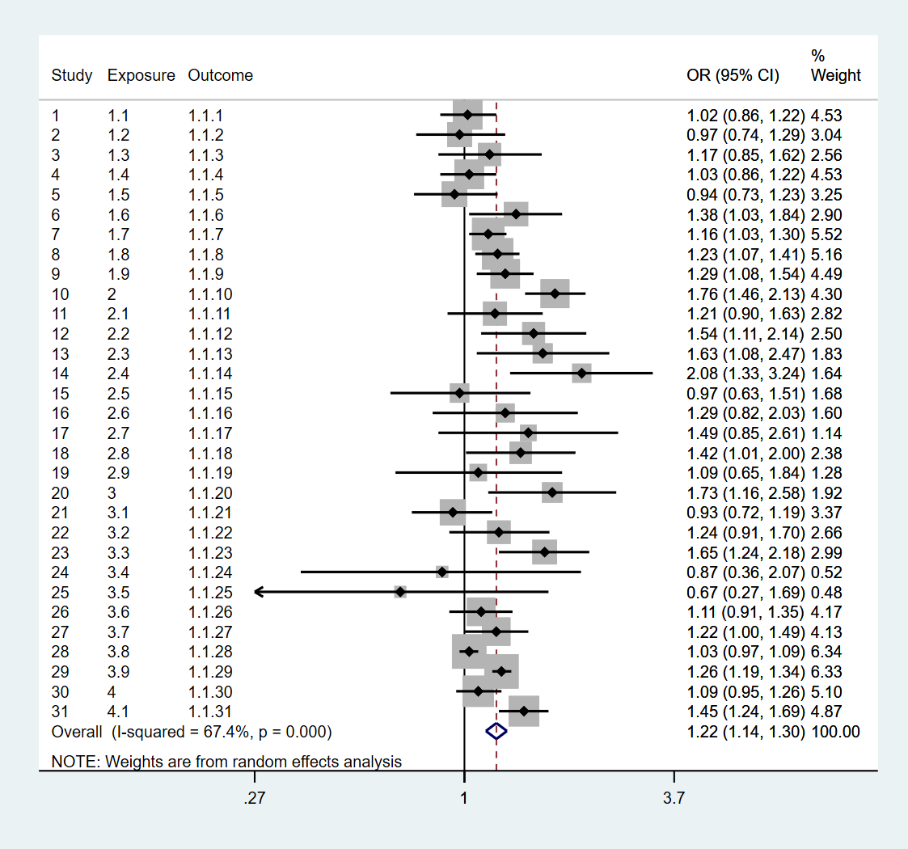** |
| Case–Control | **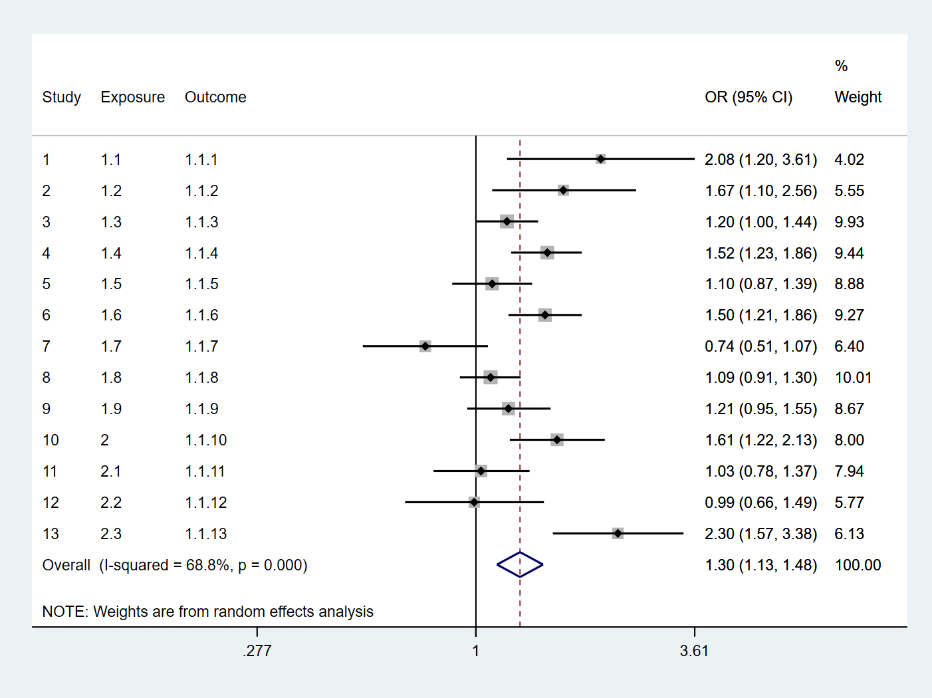** |
| **Quality grade of study** |  |
| High（NOS≥6） | **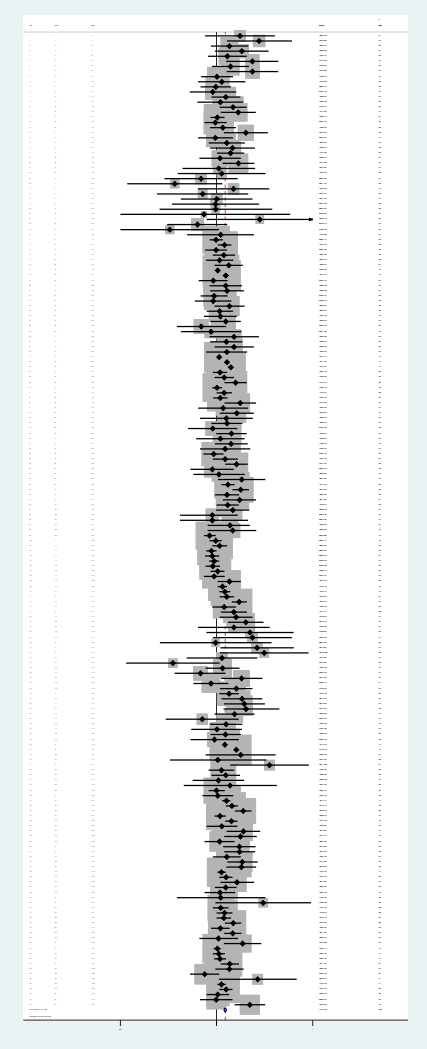** |
| Low（NOS＜6） | **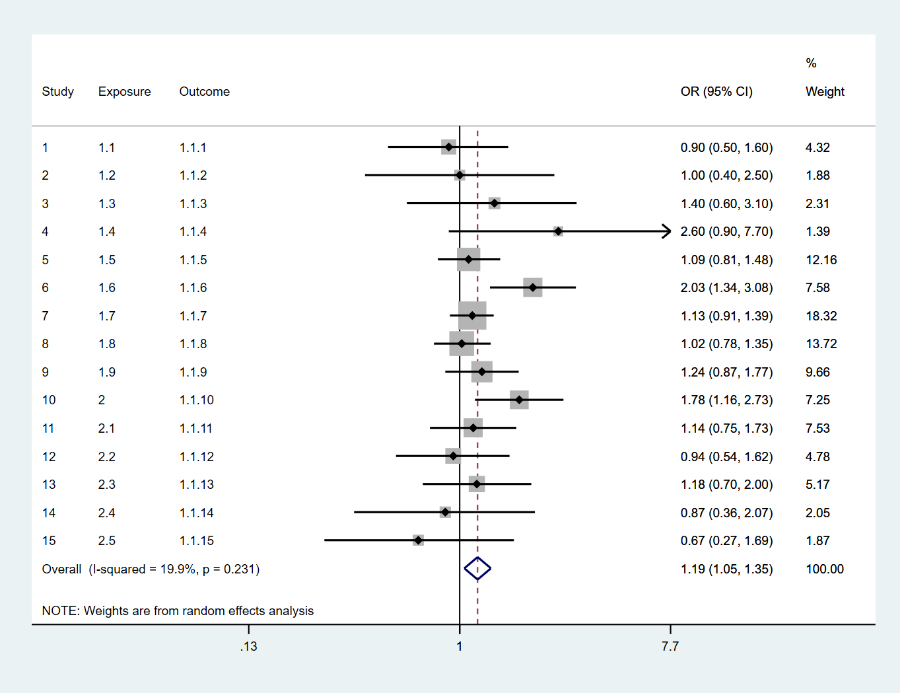** |
